# Antimicrobial Potential and Phytochemical Profile of Wild and Cultivated Populations of Thyme (*Thymus* sp.) Growing in Western Romania

**DOI:** 10.3390/plants10091833

**Published:** 2021-09-03

**Authors:** Rodica Beicu, Ersilia Alexa, Diana Obiștioiu, Ileana Cocan, Florin Imbrea, Georgeta Pop, Denisa Circioban, Cristian Moisa, Andreea Lupitu, Lucian Copolovici, Dana Maria Copolovici, Ilinca Merima Imbrea

**Affiliations:** 1 Faculty of Horticulture and Silviculture, Banat’s University of Agricultural Sciences and Veterinary Medicine “King Michael I of România” from Timișoara, Calea Aradului, 119, 300645 Timisoara, Romania; rodica.beicu@usab-tm.ro (R.B.); ilinca_imbrea@usab-tm.ro (I.M.I.); 2 Faculty of Food Engineering, Banat’s University of Agricultural Sciences and Veterinary Medicine “King Michael I of România” from Timișoara, Calea Aradului, 119, 300645 Timisoara, Romania; ileanacocan@usab-tm.ro; 3 Faculty of Veterinary Medicine, Banat’s University of Agricultural Sciences and Veterinary Medicine “King Michael I of România” from Timișoara, Calea Aradului, 119, 300645 Timisoara, Romania; 4 Faculty of Agriculture, Banat’s University of Agricultural Sciences and Veterinary Medicine “King Michael I of România” from Timișoara, Calea Aradului, 119, 300645 Timisoara, Romania; florin_imbrea@usab-tm.ro (F.I.); georgeta_pop@usab-tm.ro (G.P.); 5 Department Pharmacy, Faculty of Pharmacy, “Victor Babeş” University of Medicine and Pharmacy, Eftimie Murgu Square, 2, 300041 Timisoara, Romania; circioban.denisa@umft.ro; 6 Institute for Research, Development and Innovation in Technical and Natural Sciences, Aurel Vlaicu University, Elena Dragoi St., 310330 Arad, Romania; moisa.cristian@yahoo.com (C.M.); pag.andreea@yahoo.com (A.L.); lucian.copolovici@uav.ro (L.C.); dana.copolovici@uav.ro (D.M.C.); 7 Faculty of Food Engineering, Tourism and Environmental Protection, Aurel Vlaicu University, Elena Dragoi St., 310330 Arad, Romania

**Keywords:** *Thymus pulegioides* L., *Thymus odoratissimus* Mill., *Thymus vulgaris* L., essential oil, antimicrobial activity, chemotaxonomy

## Abstract

The purpose of this study was to analyze the chemical composition and antimicrobial activity of some thymus populations collected from five different locations in Western Romania. The chemical compositions of the essential oils (EOs) were studied through GC–MS, and the biological activities were evaluated using the microdilution method. The EO yield ranged between 0.44% and 0.81%. Overall, 60 chemical compounds were identified belonging to three chemotypes: thymol (three populations), geraniol (one population) and carvacrol (one population). *Thymus vulgaris* L. is distinguished by a high content of thymol, while species of spontaneous flora (*Th. odoratissimus* and *Th. pulegioides*) contain, in addition to thymol, appreciable amounts of carvacrol and geraniol. The antimicrobial activity of each the five oils was tested on *Staphylococcus aureus* (ATCC 25923), *Streptococcus pyogenes* (ATCC 19615), *Esherichia coli* (ATCC 25922), *Pseudomonas aeruginosa* (ATCC 27853), *Shigella flexneri* (ATCC 12022), *Salmonella typhimurium* (ATCC 14028), *Haemophilus influenzae* type B (ATCC 10211), *Candida albicans* (ATCC 10231) and *Candida parapsilopsis* (ATCC 22019). The EOs showed biological activity on Gram-positive/Gram-negative/fungal pathogens, the most sensitive strains proving to be *S. pyogenes*, *S. flexneri*, *S. typhimurium* and *C. parapsilopsis* with an MIC starting at 2 µL EO/100 µL. The species sensitive to the action of *Thymus* sp. from culture or spontaneous flora are generally the same, but it should be noted that *T. odoratissimus* has a positive inhibition rate higher than other investigated EOs, regardless of the administered oil concentration. To date, there is no research work presenting the chemical and antimicrobial profiling of *T. odoratissimus* and the correlations between the antimicrobial potential and chemical composition of wild and cultivated populations of thyme (*Thymus* sp.) growing in Western Romania.

## 1. Introduction

The spontaneous species of the *Thymus* genus emphasize great taxonomical diversity, indicating a natural evolution that allowed the adaptation to various habitats and created the premises of a divergent evolution based on a great genetic variety [1,2].

In cultures, we encounter the species *T. vulgaris* L., commonly known as culture thyme. The medicinal importance and culinary purposes of this plant have brought it to the attention of botanists over time. The species has been cultivated ever since ancient times, and it is highly recognized as a very important medicinal herb and as a spice. The aerial part of the plant (herba) presents phytotherapeutically importance, as it has stomachic, choleretic, anthelmintic, antiseptic, scarring, diuretic, antidiarrheal and antiviral effects [3,4,5].

*T. pulegioides* L., with the same medicinal properties, is frequently found in meadows in the mountainous area, especially non-calcareous, skeletal, poor soils, oak to subalpine floor [6,7]. It has several subspecies present both in Romania [7] and in Europe [8], and it is sometimes difficult to distinguish between them, even by specialists.

*T. odoratissimus* Mill. is a species with a lower range, compared to the previous species, being found in Europe mainly in the eastern part of the continent [8]. In Romania, the species vegetates in sunny meadows, from the plain to the mountains [6,7].

Thymus-based products are widely used both in human [9] and veterinary [10] medicine as cosmetic and alimentary additives [5]. The essential oil (EO) extracted from wild thyme populations is used in the food industry as a spice, in the pharmaceutical industry as an antimicrobial agent and in the medical field [11,12,13]. The chemical composition of the EO varies significantly from one species to another and sometimes even in the same species [14,15].

Previous results have demonstrated that species from *Thymus* genus are highly polymorphic [16], and more than twenty EO chemotypes were noticed in different species of the genus [17].

The reporting of its chemical composition, but also studies of flora and vegetation, require the precise determination of the species on a given field. The accuracy of the results and the correctness of the interpretation are tightly bound to taxonomical determination. Biochemical tests might offer valid answers and new instruments in taxonomical determinations, especially regarding the *Thymus* genus.

The phenolic monoterpenes represent the main constituents of EO in the species of the *Thymus* genus [18]. Among said phenolic compounds, the predominant ones are thymol and carvacrol [18,19,20]. The EOs have antioxidant [21,22], antimicrobial [23] and antifungal properties [24,25].

Scientific articles suggest that the yield and chemical composition of EOs obtained from *Thymus* species and subspecies are influenced by multiple factors such as geographic zone, climate and vegetation period [4,26]. However, based on the phenolic compounds found in the EO, the thyme populations can be classified, according to specialty studies, into several chemotypes [4,14,18].

Th literature mentions several scientific studies regarding the chemical characteristics of the spontaneous populations of wild thyme around the globe: France [14], Morocco [27], Iran [28], Serbia and Romania [29], Uzbekistan [30], etc. Among this species, *T. odoratissimus* (syn. *T. glabrescens*) and *T. pulegioides* were proven to be widely dispersed, adapting to different circumstances, and EO diversity was considerable [31].

In recent times, research in Romania has focused on cultivated species of the genus *Thymus*, especially *T. vulgaris* [32,33,34], including species of spontaneous flora, but cultivated on small areas [35], and less on species that grow spontaneously, harvested in situ from the western part of the country. Additionally, the study of antimicrobial effects on Gram-positive and Gram-negative bacteria and fungi of *Thymus* species from spontaneous flora is of interest in the context of promoting natural medicine as an alternative to synthetic antibiotics. The microorganisms used in this paper are capable of causing diseases in the human body that can sometimes be fatal. Two Gram-positive strains (*S. pyogenes* and *S. aureus*), five Gram-negative strains (*H. influenza*, *S. typhimurium*, *E. coli*, *P. aeruginosa*, *S. flexneri*) and two fungal species (*Candida parapsilosis* and *C. albicans*) were chosen. Microbes such as bacteria, molds and yeasts are employed in food production and as food ingredients, such as the production of wine, beer and bakery and dairy products. On the other hand, the growth and contamination of spoilage and pathogenic microorganisms is considered to be one of the main causes of foodstuff loss nowadays. Lorenzo et al. [36] present in their study, published in 2018, the most important bacterial and fungal strains involved in food spoilage or contamination, foodborne diseases, resistance to thermal processing and occurrence in different outbreaks, the strains selected in our research covering all these main groups.

The study is carried out with the following research directions: (i) obtaining EOs and determining their chemical composition using GC–MS; (ii) testing the biological activity of the EOs on the selected microorganisms; (iii) analyzing the existence of a correlation between the studied parameters.

## 2. Results and Discussion

### 2.1. EO Yield and Composition

**Figure 1:**
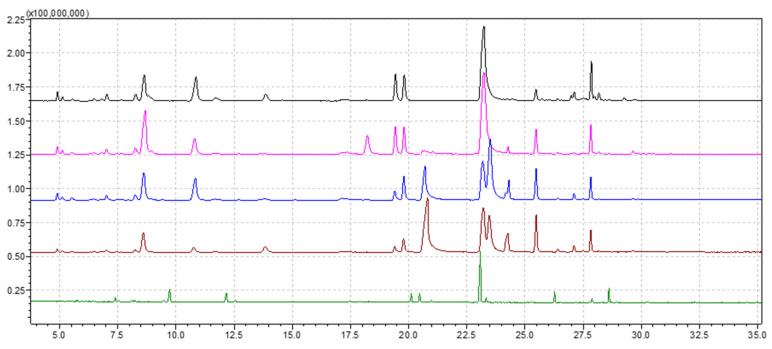
Chromatograms obtained for the EOs extracted from each thyme population: *T. odoratissimus* (TgS)—black; *T. pulegioides* (TpP)—pink; *T. pulegioides* (TpC)—blue; *T. pulegioides* (TpB)—red; *T. vulgaris* (TvL)—green.

Hydrodistillation of aerial parts of five populations of *Thymus* sp., harvested from five distinct areas of Banat, Romania, namely *T. odoratissimus* Mill. (one population), *T. pulegioides* L. (three populations) and *T. vulgaris* L. culture (one population), gave yellow and brown colored EOs, characterized by a typical odor, with a yield of 0.62%, 0.46%, 0.44%, 0.49% and 0.81% (reported by weight of dry vegetable mass) for the samples from TgS, TpP, TpC, TpB and TvL, respectively (Table 1). The overlayed chromatograms of the obtained EOs are shown in Figure 1, and the chemical composition is presented in Table 2. The individual chromatograms of each EOs are presented in Supplementary Materials S1.

In total, 60 compounds were detected (32 for TgS, 33 for TpP, 36 for TpC, 29 for TpB and 23 for TvL), phenolic compounds being the main substances in all analyzed EOs. The chemical compounds found in the EOs extracted from five thyme populations can be divided into three different chemotypes. Three EOs were characterized by high amounts of thymol (TgS–30.82%, TpP–33.81% and TvL–40.85%) and can be classified as EOs belonging to the thymol chemotype. One EO revealed carvacrol to be the main chemical compound (TpC–25.43%) and it can be classified as an EO belonging to the carvacrol chemotype, and one EO was assessed to have *cis*-geraniol as the main chemical compound (TpB), which can be classified as belonging to the geraniol chemotype. Linalool, which is considered to be responsible for antimicrobial [37,38] and antifungal activity [37] of EOs by interfering with biofilm formation and stability [39], was identified in three populations of thyme. Our results were in agreement with previous studies, which showed that *T. pulegioides* has a chemical polymorphism with different chemotypes [17,40] that show spatial segregation in nature: phenolic chemotypes (carvacrol, thymol) and non-phenolic chemotypes (geraniol, alpha-terpineol or linalool) [41,42].

Additionally, for *T. odoratissimus*, the monoterpene fraction (82.32%) was constituted by monoterpene hydrocarbons (25.92%), among which the main representatives were *p*-cymene (8.55%) and γ-terpinene (8.96%). Among oxygen-containing monoterpenes (56.4%), linalool (2.84%) was the most abundant. Germacrene D (1.88%) was the main component of the sesquiterpene fraction. Furthermore, several phenolic monoterpene compounds (51.77%) with high biological importance were found to have significant prevalence values, namely thymol (30.82%), carvacrol (7.94%), carvacrol methyl ether (6.67%) and thymol methyl ether (6.34%).

The EO obtained from *T. pulegioides* in Prigor (TpP) was mainly constituted by thymol (33.81%). The monoterpene fraction (89.36%) comprised hydrocarbons (27.03%) with *p*-cymene (15.12%) as the predominant component. Among oxygen-containing monoterpenes (62.33%), although thymol was the most abundant compound (33.81%), the presence of α-terpineol (6.29%) is also important to stipulate. Caryophyllene (4.19%) and aromadendrene (4.14%) represented the main sesquiterpene hydrocarbons.

In the *T. pulegioides* EO from Carasova (TpC), among the compounds found within the monoterpene fraction, carvacrol (25.43%) and thymol (13.93%) were the most abundant ones. Regarding the monoterpene fraction, a significant difference was determined. As such, the monoterpene hydrocarbons were found to have a relatively high prevalence (21.50%), among which the main representatives were *m*-cymene (8.45%) and γ-terpinene (7.47%). The oxygen-containing monoterpenes (67.13%), among which the main non-phenolic representatives were *cis*-geraniol (13.12%) and *cis*-geranyl acetate (3.33%), were present in a significant percentage. Isocaryophyllene (5.22%) was the main component of the sesquiterpene fraction of the EO, followed by germacrene D (1.05%).

The EO from *T. pulegiodes* collected from Bazias (TpB) presented a different composition. In this EO, monoterpenes constituted the most abundant fraction (86.35%), with a prevalence of monoterpene hydrocarbons (10.46%) and oxygen-containing monoterpenes (75.89%), with a great amount of *cis*-geraniol (28.35%), as well as the phenols thymol (17.14%) and carvacrol (15.21%). Among sesquiterpenes, only isocaryophyllene (6.83%) was present in an appreciable amount.

The three studied EOs from *T. pulegioides* were quite different regarding the relative percentage of the main components; thymol was predominant in *T. pulegioides* collected from Prigor, while carvacrol and geraniol were only 5.71% and 1.88%, respectively. Moreover, carvacrol is the main phenolic compound in *T. pulegioides* from Carasova, and geraniol was the predominant compound in *T. pulegioides* from Bazias. These differences in the EO of *T. pulegioides* confirmed that the species of the genus *Thymus* are taxonomically and genetically complex [37]. Previous papers on the EOs of *T. pulegioides* concluded that there is no clear chemical relation between different varieties of this species and chemotypes [43], so it is recommended and useful to expand this study area regarding *Thymus* genus.

The EO of *T. vulgaris*, the cultivated species of thyme, collected from Lovrin, was characterized by high percentages of thymol chemotype (40.85%). The monoterpene hydrocarbons were quite similar to the other EOs (23.40%). *p*-Cymene (10.94%) and γ-terpinene (8.50%) were the main compounds, while the oxygenated monoterpenes represented 55.36%. Caryophyllene (5.98%) was the main component of the sesquiterpene fraction of the EO, followed by germacrene D (2.24%).

Therefore, six chemotypes were identified in a culture of *T. vulgaris* in France, based on the diversity of the EOs (geraniol, linalool, α-terpineol, thuyanol-4, thymol and carvacrol), with stable specific characters in both natural habitats and experimental cultures, which can also be passed on via inheritance [14]. These ”chemical species” are considered intraspecific, the species being homogenous by morphologic and karyotypical criteria, and the distribution frequency of these chemotypes is due to the ecological and genetic factors, especially climatic factors [14]. The presence of some phytochemical chemotypes has been reported in other species of the *Thymus* genus as well [4,26].

Among this species, *T. odoratissimus* (syn. *T. glabrescens*) and *T. pulegioides* were proven to be widely dispersed, adapting to different circumstances, and the EO diversity was considerable [31].

For example, *T. pulegioides* has been characterized by a highly polymorphic chemical level [16,40]. Previous results have demonstrated that this species is polymorphic [16], and eight chemotypes have been determined [17,36]. The thymol/carvacrol chemotype is one of the most frequent in Italy [41].

### 2.2. Antimicrobial Activity

**Figure 2:**
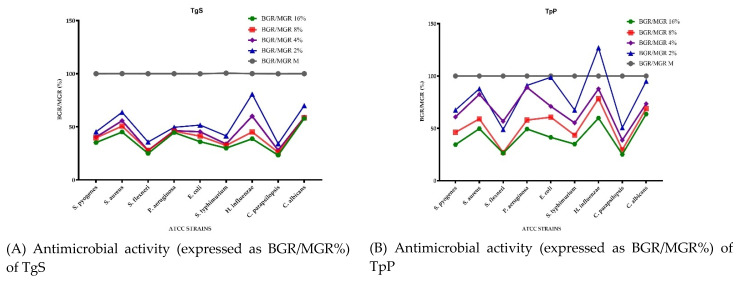

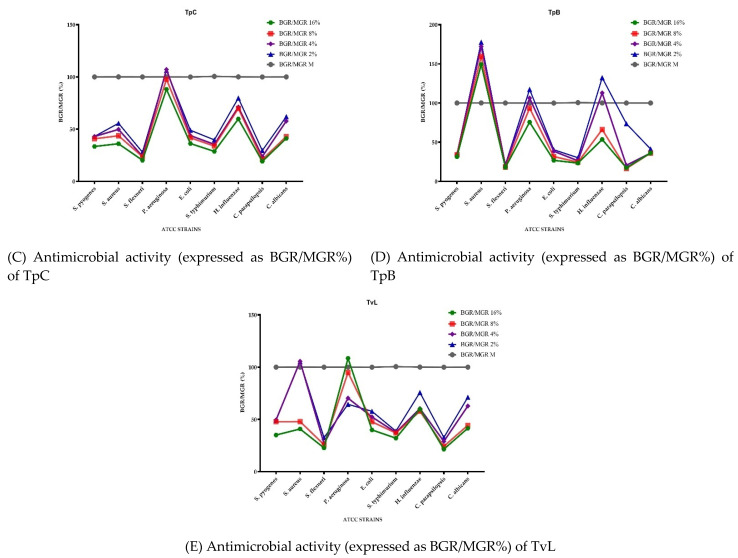
Antimicrobial activity of analyzed EOs expressed as BGR/MGR (%): (**A**) TgS; (**B**) TpB; (**C**) TpC; (**D**) TpP; (**E**) TvL.

**Figure 3:**
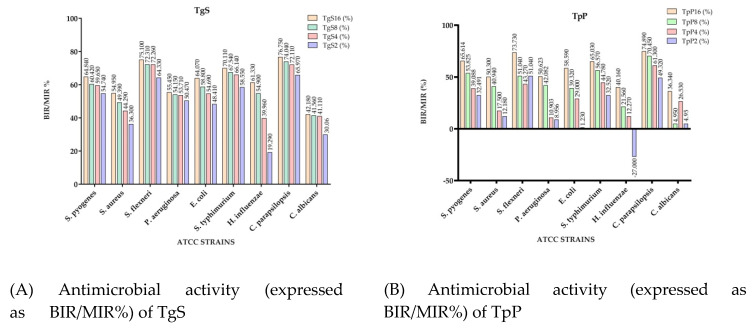

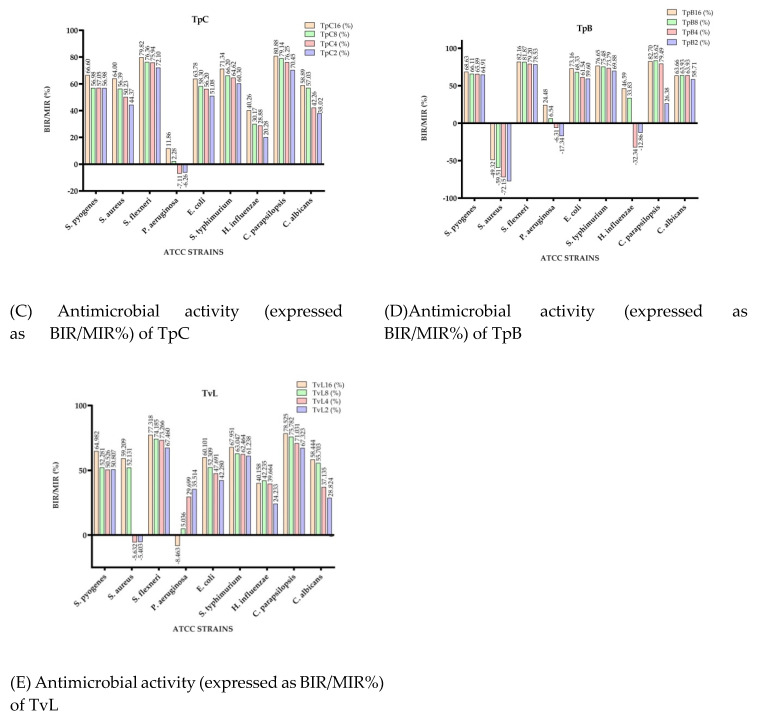
Antimicrobial activity of analyzed EOs expressed as BIR/MIR (%): (**A**) TgS; (**B**) TpB; (**C**) TpC; (**D**) TpP; (**E**) TvL.

Figure 2 presents the microdilution method results of the EOs tested, expressed as the bacterial growth rate (BGR %), calculated according to Formula (1), and mycelial growth rate (MGR %), calculated according to Formula (3), both defined in the Materials and Methods section. Figure 3 shows the bacterial inhibition rate (BIR %)/mycelial inhibition rate (MIR %) calculated according to Formulas (2) and (4), defined in the Materials and Method section. All values presented below the baseline in Figure 3 represent a strain-boosting effect. Table 3 presents the optical density (OD) of EOs at different concentrations applied on the selected bacterial and fungal strains, as is described in Section 3.4. The MIC (µL EO/100 µL) values are presented in Table 4.

Reported as BGR/MGR%, the microbialgrowth rate is the growth of the bacterial/micelial mass, where growth was identified by spectophotometric measurement, while BIR/MIR% represents the bacterial/micelial wheremass loss after the inhibitory effect of EOs, was registered through spectrophotometric measurements. Concerning the effect of the tested EOs on the bacterial/fungal strains, the registered effect is either a strain-boosting effect, where the bacterial/micelial mass growth of the strain affected by the oil is larger than that found in the case of the simple strain, which is therefore a potentiating effect, or an inhibitory effect, in which case the mass growth of the bacterial/micelial strain is lower than that reported for the simple strain.

Concerning the antibacterial activity of TgS, the evolution is in close correlation with the concentration used, TgS presenting a general inhibitory activity. The most affected strains were *S. flexneri*, *S. typhimurium* and *S. pyogenes*. For *S. flexneri* the growth rate (BGR), depending on the concentration, varied from 24.90% to 35.67% (Figure 2A), with an inhibition rate (BIR%) ranging from 64.33% to 75.10% compared to the negative control (Figure 3A). BGR% ranged between 29.89% and 41.45% (with an inhibitory percentage ranging from 58.55% to 70.11% BIR) in the case of *S. typhimurium*. The inhibitory effect of TgS was also detected when other bacterial strains were tested, but with a lower inhibition rate.

Regarding the antifungal activity, the evolution is also connected to the concentration, and *C. parapsilopsis* proved to be more sensitive to TgS compared to *C. albicans*. The MGR% of *C. parapsilopsis* was 23.25% and 34.03% when TgS 16% and TgS 2%, respectively, were applied, while the MGR% of *C. albicans* ranged between 57.82 and 69.94%. The inhibitory effect was proved by MIR%, with values ranging from 30.06% to 42.18% in the case of *C. albicans* and 65.97–76.75% for *C. parapsilopsis*. As an overview, for all the tested strains, TgS MIC proved to be the 2 µL EO/100 µL tested concentration, MIC being the lowest compound concentration that yields no visible microorganism growth compared to the control (Table 4). The results of our study were in agreement with the study of Karpiński et al. (2020), on *T. vulgaris*, in which the MIC value against *C. albicans* was found between 0.16 and 313 µL/mL [24].

Comparing the BGR percentages, the most sensitive ATCC strains, when TpP is concerned, were *S. flexneri*, *S. pyogenes* and *S. typhimurium (*Figure 2B). For *S. flexneri* the growth rate (BGR), depending on the concentration, varied from 26.27% to 48.96% (Figure 2B), with an inhibition rate (BIR%) ranging from 51.04% to 73.73% compared to the negative control (Figure 3B). Against *S. pyogenes* the BIR was found between 32.49% and 65.61% and between 32.52% and 65.03% when the tested strain was *S. typhimurium.* The concentration influence trend was similar for the other sensitive strains, with BIR in the range of 12.18–50.30% against *S. aureus* and between 1.23% and 58.59% against *E. coli*.

Concerning TpP, our results are in agreement with previous studies in which de Martino et al. [41] showed a sensitivity that increased with the concentration. The concentrations tested through disk diffusion against *S. aureus*, *E. coli* and *P. aeruginosa* were found to be in the 0.62–10 mg/mL range. In our case, the MIC values were determined at 4 µL/100 µL for *H. influenzae* and 2 µL/100 µL for the rest of the tested strains. *H. influenzae* proved to be the only ATCC strain in which the inhibitory effect was not present at a 2% tested concentration, the evolution of BIR%, in this case, being negative, therefore proving a boosting bacterial effect with a −27% value. The growth rate for *H. influenzae* was concentration-dependent, ranging from 59.84% up to 127% (Figure 2B).

In terms of antifungal activity, the inhibition rate showed a better effect on *C. parapsilopsis* than on *C. albicans*, values being different by 40%. The less effective antifungal effect against *C. albicans* was demonstrated by the MGR% values, which ranged from 95.05% for TpP tested in a concentration of 2% to 63.66% in the case of TpP 16% (Figure 2B).

The results presented in Figure 2C and Figure 3C showed that the best antimicrobial effect recorded by TpC (TpC 2%, TpC 4%, TpC 8% and TpC 16%) were against *S. flexneri* (with BGR ranging from 27.90% to 20.18% and BIR 72.10–79.82%), *S. typhimurium* (with BGR values of 28.66–39.70% and BIR values of 60.30–71.34%) and *S. aureus* (with BGR ranging from 36% to 55.63% and BIR from 44.37 to 64%). The inhibitory effect of TpC against *S. pyogenes* varied to a small extent with the concentration. The obtained values of BGR were 33.40% for TpC applied at a concentration of 16% and 43.20% in the case of TpC 2%, and BIR ranging from 56.98% to 66.60%, depending on the concentration. In this context, Afonso et al. (2018), reported a MIC value of 1.13 µL/100 µL for *T. pulegioides* against *S. aureus* and 5.75 µL/100 µL against *S. typhimurium* [43]. These results are also in agreement with the data presented by Vaičiulytė et al. that demonstrated in their research (2021) a MIC of 31.3 µg/mL against *E. coli* and *S. aureus* [44].

TpC had a proven growth-boosting effect at 2% and 4% concentrations tested against *P. aeruginosa* with MGR% values of 107.11% for TpC 4% and 106.26% in the case of TpC 2%. The inhibitory effect started at 8% concentration, data in correlation with the research done by Afonso et al. (2018), which presented in their study a 5.75 µL EO/100 µL value as MIC against *P. aeruginosa* [43].

To summarize the data regarding the antifungal activity, TpC was effective and inhibited both fungal strains. MIR% ranged in the case of the *C. parapsilopsis* strain from 26.38% to 82.70%, and from 58.71% to 63.66% in the case of *C. albicans*. Our results were in agreement with previous studies [24], which presented 0.8 µL/100 µL as MIC against *Candida* sp., values similar to our findings.

Concerning the antibacterial effect of TpB, the highest inhibitory activity was proved to be against *S. flexneri*, when the BGR% varied from 17.83% for 16% concentration, to 21.47% for 2% concentration compared to the control, and BIR% ranged between 78.53% and 82.16% (Figure 2D and Figure 3D). A high inhibitory effect was also proved against *S. typhimurium*, with BIR% varying from 68.88% to 76.65%. The evolution was connected to the tested concentration, as BGR% ranged from 69.88% for the concentration 2% of TpB up to 76.65% for TpC at 16% concentration. The 2 µL/100 µL concentration proved to be MIC for all the tested microorganisms except for *S. aureus*, *P. aeruginosa* and *H. influenzae* (Table 4).

Against *S. aureus*, TpB produced a strain boosting effect with BGR% values of 177.54% for TpB 2% and 149.31% for TpB 16%, the BIR% values being negative. Our results were in agreement with previous studies, which showed that the antimicrobial effect of EO on *T. marschallianus* is high on Gram-positive bacteria, except *S. aureus* [45].

The strain boosting effect was also shown in the case of *P. aeruginosa* and *H. influenzae* at 2% and 4% concentrations tested. For *P. aeruginosa*, the inhibitory effect had a slower evolution with BIR% of 6.54% at 8% and 24.48% for TpB 16%. Concerning the TpB activity against *H. influenzae*, the effect was shown at 8% concentration with BGR% of 66.72% and 53.41% in the case of TpB 16%.

Regarding the antifungal activity of TpB, *C. parapsilopsis* presented a higher percentage of fungal mass loss compared to *C. albicans* with MIR% ranging from 26.38% to 82.70%. From a different point of view, *C. albicans* proved to be more inhibited at a lower concentration, with values of MIR% starting from 58.71% at 2% up to 63.66% for 16%, compared to the values obtained against *C. parapsilopsis*.

To summarize the data regarding the TvL antimicrobial activity presented in Figure 2E and Figure 3E, we can observe that TvL was most effective against *S. flexneri, S. typhimurium* and *S. pyogenes* and both fungal strains. Concerning the highest antibacterial activity proved by BIR%, the obtained values, at 16% concentration, ranged between 64.94% and 77.32% (Figure 3E). Regarding the other tested strains, *E. coli* and *H. influenzae*, TvL had a proven growth-inhibitory effect, with a BGR compared to control (%) varying from 39.90% to 57.72% for *E. coli* and from 59.82% to 75.77% for *H. influenzae.* Our results were in agreement with previous studies [45,46,47], which showed that *T. vulgaris* EO has an inhibitory effect, especially on Gram-positive bacteria.

The results were in correlation with the findings presented by Al-Shuneigat et al., which stated inhibitory results for concentrations varying from 0.5% to 4% [48].

Concerning MIR%, the effect was also an inhibitory one, with values varying from 67.32% to 78.52% for *C. parapsilopsis* and 28.82% to 58.44% for *C. albicans*. Our results were in agreement with previous studies [49], which showed a strong antifungal activity of the EO in *T. vulgaris* and a correlation of this effect with the amount of thymol, *p*-cymene and γ-terpinol in this oil.

In the case of *S. aureus*, the inhibitory effect was proved at 8% with BIR% of 52.13%, while the lowest concentrations tested proved a growth-boosting effect, with BGR% values compared to control of 105%. The effect on *P. aeruginosa* proved to be one of mass growth in close correlation with the concentration, as the BIR% values started at 35.51% for 2% concentration and decreased at −8.46% for TvL16%. This result was inconsistent with Mancini et al. (2015), but because significantly lower concentrations of EO were used, further studies are needed to clarify the dynamics of the antimicrobial effect for this EO [46].

de Carvalho et al. [50] presented, in their study published in 2015, a MIC value of 25 µL EO/100 µL against *S. aureus*, a value much higher than the one obtained by us (Table 4).

Analyzing the complete results of the antibacterial tests, we can conclude that the most affected strains when *Thymus* sp. EOs were used were *S. flexneri*, *S. typhimurium* and *S. pyogenes*, and regarding the antifungal activity, *C. parapsilopsis* proved to be more sensitive compared to *C. albicans*.

Our results were in agreement with previous studies [45,46,51], which showed that *Thymus* EO has in vitro inhibitory activity against some of the microorganisms tested. Using the MIC method, the lowest inhibitory concentrations were found to be against *S. enteritidis*, *P. aeruginosa* and biofilm-forming bacteria. Galovičová et al. (2021) highlighted that the highest inhibitory concentrations of *T. serpyllum* were identified against *S. aureus* and *C. tropicalis*, and the lowest inhibitory concentrations were found against *S. enteritidis* and *P. aeruginosa* [52]. Earlier studies have demonstrated the antifungal potential of thymol against *Candida* species, because this interferes with the formation and viability of mycelium hyphae [25,49].

The analysis of correlation between the antimicrobial effect of *Thymus* species against the analyzed strains and the chemical composition is presented in the Supplementary Materials file 2.

TgS represents the least studied *Thymus* species from spontaneous flora, in terms of antimicrobial effects and their correlation with the chemical composition. The results reported in Table 2 highlight an important diversity in terms of the chemical composition of TgS. We noticed a high content of thymol (30.82%), but also a significant percentage of *p*-cymene (8.55%), carvacrol (7.94%) and γ-terpinene (8.96%). The antimicrobial activity of TgS was the most important, compared to the other samples analyzed, and refers to the preponderant inhibition of *S. pyogenes*, *S. typhimurium* and *C. parapsilopsis* strains. Supplementary Materials file 2 shows that the strongest positive correlations were reported for the mentioned chemical compounds, namely the pairs *S. pyogenes*/*p*-cymene (r = 0.904) and γ-terpinene (r = 0.852); *S. typhimurium* and carvacrol (r = 0.999), thymol (r = 0.716), *p*-cymene (r = 0.982) and γ-terpinene (r = 0.997); and *C. parapsilopsis*/carvacrol (r = 0.957), *p*-cymene (r = 0.866) γ-terpinene (r = 0.916) and thymol (r = 0.905), which confirms that some minor compounds, or the association of minor compounds with major compounds in the EO’s composition leads to strong antimicrobial effects as fewer individual compounds, even if they are found in large quantities.

In this regard, Mancini et al. (2015) highlighted that minor components have a critical part to play in antibacterial activity, possibly by producing a synergistic effect between other components [46]. Ahmad and coworkers (2014), reported that synergistic and additive interactions occur between the major and minor constituents present in the EO of *Thymus* and, in this way, the antimicrobial efficacy could be enhanced [51].

Additionally, Guimarães et al. (2019) stated that the classification of the antimicrobial actions of pure compounds is not well consolidated in the literature, making difficult the comparison with previous results. They proved that thymol had a positive effect on *Salmonella* sp., *E. coli* and *S. aureus*, and that oxygenated functional groups in terpene compounds exhibited better antimicrobial activity than hydrocarbons [53].

The correlation data of TpP presented in Supplementary Materials file (Table S1) are in agreement with the results regarding the chemical composition (Table 2) and the antimicrobial effects (Figure 2B and Figure 3B). TpP is characterized by an important content of *p*-cymene (15.12%) and *cis*-geraniol (1.88%), in addition to thymol, which is the major compound in all *Thymus* species analyzed (Table 2), as well as high inhibitory effects on the development of stem cells *S. flexneri*, *S. pyogenes*, *S. typhimurium* and *C. parapsilopsis* (Figure 2B and Figure 3B). The existence of a strongly positive correlation between *S. pyogenes* and *p*-cymene (r = 0.925), *S. flexneri* and *p*-cymene (r = 0.989), *S. typhimurium* and *cis*-geraniol (r = 0.945) leads to the conclusion that the synergism generated by the association of *p*-cymene with *cis*-gernaiol could be responsible for the antibacterial activity on the mentioned strains and not necessarily the presence of thymol, which registers negative correlations with bacterial strains. Marchese et al. (2014) stated that *p*-cymene, a major compound, exerted good antibacterial activity against *S. pyogenes* and methicillin-resistant *S. aureus* (MRSA) and also presented the possibility that *p*-cymene can enhance the inhibitory effects of carvacrol when the two compounds are used together [54].

The nature of strains could be responsible for synergistic or antagonistic effects. Synergistic interactions were mostly observed with Gram-positive microorganisms, while yeasts and Gram-negative strains showed weak synergistic interactions [51].

Regarding the antifungal activity associated with *C. parapsilopsis* strains, it seems to be associated with oxygenated terpenes, such as *cis*-geraniol (r = 0.945). In this regard, the results reported by Sokovic et al. (2009), regarding the investigation of antifungal properties of *Thymus* sp., showed that hydrocarbon monoterpenes had the lowest antifungal activity, and a larger antifungal potential could be due to the presence of oxygenated terpenes or of those with phenolic structures [49].

The analysis of correlations between the antimicrobial effects of TpC against the analyzed strains and the chemical compositions highlighted that strong (r > 0.7) positive correlations were recorded between pairs, suggesting possible antimicrobial activity generated by these chemical components: *S. pyogenes* and carvacrol (r = 0.999) and *cis*-geraniol (r = 0.800); *S. aureus* and *p*-cymene (r = 0.866) and *cis*-geraniol (r = 0.741); *S. flexneri* and *p*-cymene (r = 1.000) and *cis*-geraniol (r = 0.977); *E. coli* and carvacrol (r = 0.997) and *cis*-geraniol (r = 0.821); *S. typhimurium* and carvacrol (r = 0.784); *H. influenzae* and carvacrol (r = 0.976), *p*-cymene (r = 0.778) and *cis*-geraniol (r = 0.893); and *C. parapsilopsis* and *p*-cymene (r = 0.756).

Togashi et al. (2008) discussed the antibacterial activity and the mode of action of farnesol against *S. aureus*, when geraniol was added to a bacterial suspension. The authors assumed that geraniol increased the growth-inhibitory activity of farnesol. Their results revealed that terpenes might interact with each other and with bacterial cells to increase or decrease the antibacterial activity of each other [55].

Another study highlighted that geraniol significantly increased the efficacy of chloramphenicol by targeting efflux mechanisms and produced a significant restoration of susceptibility of the multidrug resistance strains tested [56].

In the case of TpC, a greater number of positive correlations were observed between the chemical composition and the antimicrobial activity. In addition to the associations mentioned in the case of the other EOs analyzed, there was a positive correlation of *E. coli* and carvacrol (r = 0.997) and *cis*-geraniol (r = 0.821). TpC represented the sample with the highest percentage of carvacrol (25.43%), 13.12% *cis*-geraniol and a high *E. coli* inhibition rate (68.78%), which suggest a possible association of the antibacterial effect on *E. coli* with the presence of the mentioned chemical compounds. Other studies have associated *E. coli* inhibition with the chemical composition of *Thymus* EO [51]. Ahmad et al. (2014) suggested that the monoterpene phenols, thymol and carvacrol, were found to be the most active constituents against *E. coli,* while linalool were generally found to have moderate activity, and *p*-cymene, borneol, α-terpinene and γ-terpinene exhibited weak antimicrobial activity [51].

The correlation analysis of TpB indicates a possible antibacterial effect against *E. coli* generated by the synergism achieved by associating several chemical components such as carvacrol (r = 0.995), thymol (r = 1.000), γ-terpinene (0.924), *cis*-geraniol (r = 0.995) and linalool (r = 0.901).

TvL is the most studied species in terms of antimicrobial effects and association with chemical composition. Thymol is the major chemical compound (40.85%) of the EO and defines the biologically active behavior of TvL. The analysis of correlation data shows that thymol correlated positively with *S. flexneri* and *S. aureus* (r = 0.771). Other authors reported the antimicrobial, antibiofilm and biochemical properties of *Thymus* EO against clinical isolates of opportunistic infections [57,58].

The analysis of correlation between the antimicrobial effect of TvL against analyzed strains and the chemical composition proved a moderate (r > 0.5) positive correlation between *C. albicans* and carvacrol (r = 0.663).

Other strong (r > 0.7) positive correlations were recorded between pairs, suggesting possible antimicrobial activity generated by these chemical components: *S. aureus* and thymol (r = 0.771); *S. flexneri* and thymol (r = 0.771); *E. coli* and thymol (r = 0.817); *C. parapsilopsis* and *p*-cymene (0.945) and γ-terpinene (r = 0.938); and *C. albicans* and *p*-cymene (0.756) and γ-terpinene (r = 0.770). The cumulative correlation data for all *Thymus* species studied indicate that for the same chemical compound and the same microbial strain, the correlation between the parameters may be different, depending on the studied species. This fact confirms what was noticed by other authors [51], namely that the complexity of the chemical composition and especially the minor components can generate synergistic or antagonistic effects, so that in complex biological systems a mathematical prediction is difficult to achieve.

The different antimicrobial activities of these oils might be due to the small variation in their chemical profile. It was reported in the literature that various chemical compounds have direct activity against many species of bacteria, such as terpenes and a variety of aliphatic hydrocarbons (alcohols, aldehydes and ketones). The lipophilic character of their hydrocarbon skeleton and the hydrophilic character of their functional groups are of main importance in the antimicrobial action of the components of EOs, and the importance of the hydroxyl group of phenolic structures has been confirmed [51].

To conclude the correlation study, we can state that the chemical compounds found in the EOs extracted from *Thymus* sp. are responsible for the antimicrobial activity, the existing correlations being influenced by the synergism/antagonism exerted between the compounds and the morphological characteristics of the strains (Gram-positive, Gram-negative and fungi).

## 3. Materials and Methods

### 3.1. Plant Material

The species analyzed in the present study are presented in Table 5. *T. odoratissimus* Mill. (syn *T. glabrescens* Willd.), collected around the settlement of Silagiu (code: TgS), *T. pulegioides* L., collected around the settlement of Prigor, (code: TpP), *T. pulegioides* L., collected around Carasova (code: TpC), *T. pulegioides* L., collected around Bazias (code: TpB) and *T. vulgaris* L., cultivated and collected from the Agricultural Research and Development Station Lovrin (code: TvL).

The analyzed *Thymus* populations were harvested during flowering, from five different habitats in Banat, Romania, in 2019 (*T. pulegioides*, *T. vulgaris*) and 2020 (*T. odoratissimus*). These were studied in the laboratory to establish the species accurately. The determination was made with the help of specialized determinants [7,8], and the scientific names were updated after The EuroPlusMedPlantbase (electronic edition) [9]. Species were determined using morphological criteria described in the literature [3].

Sample vouchers for each population were stored after identification in the herbarium of the Crop Science Department within the University of Agricultural Sciences and Veterinary Medicine of Banat “King Mihai I of Romania” from Timisoara.

### 3.2. Obtaining the EOs

The EOs analyzed in the present paper were obtained from mixtures of subspecies (10–20) of the same species belonging to the genus *Thymus*, from a collection area. Therefore, oils with different characteristics were obtained from the five collection areas. The extraction of EOs was carried out from the dried aerial parts of thyme plant, using hydro-distillation equipment (Clevenger extractor, experimental model, Timisoara, Romania). The resulting EO and aromatic water mixture were separated using a separating funnel. The pure EOs were stored in glass vials at +4 °C until further analysis. The extraction yield was calculated as a percentage of dried plant herba.

### 3.3. GC–MS Analysis of EOs

The chemical constituents of EOs isolated from the aerial part of *Thymus* sp. were determined by using a gas chromatograph (Shimadzu 2010, Kyoto, Japan) coupled with a triple quadrupole mass spectrometer (TQ 8040, Shimadzu, Kyoto, Japan). The used column was an optima 1MS + WAX column (30 m × 0.25 mm i.d., 0.25 µm film thickness, Macherey–Nagel, Duren, Germany) using helium as carrier gas at 1 mL min^−1^ flow. The oven temperature was initiated at +70 °C for 11 min and raised to +190 °C at a rate of 5 °C min^−1^ and then to +240 °C at a rate of +20 °C min^−1^, where it was kept for 5 min. Injector and MS source temperatures were set to +250 °C and +200 °C, respectively. The samples were diluted before injection (1:25, v:v), and the injection volume was 1 µL, with a split ratio of 10:1.

The EO constituents were identified based on the comparison of their retention indices (abbreviated RI^a^), determined relative to the t_R_ values of *n*-alkanes (C10–C35) on both capillary columns with those in the literature [59] and their mass spectra with those of standard compounds (α-pinene; sabinene; β-pinene; β-myrcene; α-phellandrene; 3-carene; D-limonene; *cis*-β-ocimene; *trans*-β-ocimene; carvacrol; caryophyllene) available in our laboratories or those listed in the NIST 14 and Wiley 09 mass spectral libraries (abbreviated RI ^b^).

### 3.4. Determination of Antimicrobial Activity

The microbial strains used in this study were obtained from the culture collection of the Microbiology Laboratory of the Interdisciplinary Research Platform within the University of Agricultural Sciences and Veterinary Medicine “King Mihai I of Romania” in Banat, Timisoara. In our laboratory, ATCC strains are maintained at −50 °C.

The EOs were tested against *S. aureus* (ATCC 25923), *S. pyogenes* (ATCC 19615), *E. coli* (ATCC 25922), *P. aeruginosa* (ATCC 27853), *S. flexneri* (ATCC 12022), *S. typhimurium* (ATCC 14028), *H. influenzae* type B (ATCC 10211), *C. albicans* (ATCC 10231) and *C. parapsilopsis* (ATCC 22019).

The MIC is defined as the lowest compound concentration that yields no visible microorganism growth. The method of MIC determination based on the microbial mass loss by measurement of OD by spectrophotometry according to ISO 20776-1:2019 was described in our previous research [60].

#### 3.4.1. Bacterial Culture

A 10^−3^ dilution of the fresh culture was used to perform the assay, an inoculum equivalent to a 0.5 McFarland standard. The ATCC strains were revived by overnight growth in Brain Heart Infusion (BHI) broth CM1135 (Oxoid, Hampshire, UK) at 37 °C and subsequently passed on BHI agar (Oxoid, CM1136) for 24 h at 37 °C. The cultures were then diluted at an optical density (OD) of 0.5 McFarland standard (1.5 × 10^8^ UFC×mL) using BHI broth and a McFarland densitometer Grand-Bio (Fisher Scientific, Loughborough, UK). The suspensions were tested using a 96 microdilution well plate. Using a Calibra digital 852 multichannel pipette, 100 μL of microbial suspension was placed in each well. The EOs were tested at a concentration of 2%, 4%, 8% and 16%, added in each well. The plates were covered and left 24 h at 37 °C. After 24 h, the OD was measured at 540 nm using an ELISA reader (BIORAD PR 1100, Hercules, CA, USA). Triplicate tests were performed for all samples. The suspensions of strain and BHI were used as a negative control.

To interpret the results, two indicators were calculated, namely BGR and BIR, by using the following formulas:

(1) $$\mathrm{BGR}=\frac{{\mathrm{OD}}_{\mathrm{sample}}}{{\mathrm{OD}}_{\mathrm{negative}\mathrm{control}}}\times 100(\%)$$

BIR = 100 − BGR (%)(2)

where OD sample—optical density at 540 nm as the mean value of triplicate readings for EOs in the presence of the selected bacteria; OD negative control—optical density at 540 nm as the mean value of triplicate readings for the selected bacteria in BHI.

BGR/MGR indicators are calculated values expressed as percentages of growth or inhibition, indicators used previously in several other research articles [56,60,61,62].

#### 3.4.2. Fungi Culture

A 10^−2^ dilution of the fresh culture was used to perform the assay, an inoculum equivalent to a 0.5 McFarland standard. The ATCC fungal strains were revived by overnight growth in Brain Heart Infusion (BHI) broth CM1135, (Oxoid, Hampshire, UK) at 37 °C and subsequently passed on BHI agar (Oxoid, CM1136) for 48 h at 37 °C. The cultures were then diluted at an OD of 0.5 McFarland standard using BHI broth, a value determined by using a McFarland densitometer Grand-Bio (Fisher Scientific, Loughborough, UK). The suspensions were tested using a 96 microdilution well plate by placing 100 μL of microbial suspension in each well. The EOs were tested at a concentration of 2%, 4%, 8% and 16%, added in each well. The plates were covered and left for 48 h at 37 °C. After 48 h, the OD was measured at 540 nm. Triplicate tests were performed for all samples.

To interpret the results, two indicators were calculated, namely MGR and MIR, using the following formulas:

(3) $$\mathrm{MGR}=\frac{{\mathrm{OD}}_{\mathrm{sample}}}{{\mathrm{OD}}_{\mathrm{negative}\mathrm{control}}}\times 100(\%)$$

MIR = 100 − MGR (%)(4)

where OD sample—optical density at 540 nm as the mean value of triplicate readings for EOs in presence of the selected fungi; OD negative control—optical density at 540 nm as the mean value of triplicate readings for the selected fungi in BHI.

### 3.5. Statistical Analysis

All determinations were made in triplicate, and the results are reported as mean values ± standard deviation (SD). These were calculated using GraphPad Prism (v.5.0 software, San Diego, CA, USA). The differences between means were analyzed with a one-way ANOVA, followed by a multiple comparison analysis using the *t*-test (two-sample assuming equal variances). The differences were considered significant when *p*-values < 0.05. Correlations between variables were performed using Microsoft Excel 2013.

## 4. Conclusions

The results reported here may help to shed light on the complex chemotaxonomy of the genus *Thymus*. There is a significant relationship between the antimicrobial activity of *Thymus* EOs and the presence of phenolic compounds, such as thymol and carvacrol. The correlation study highlighted that the chemical compounds of the *Thymus* sp. EOs are responsible for the antimicrobial activity, the existing correlations being influenced by the synergism/antagonism exerted between the compounds and the morphological characteristics of the strains (Gram-positive, Gram-negative and fungi).

Regarding the general results of the study, the studied EOs from *Thymus* sp. can find practical uses in preventing bacterial or fungal infections in plants, animals or humans, being 100% natural and non-toxic in small amounts.

On the other hand, significant variations in the chemical profile identified in *T. pulegiodes* collected from different locations in Western Romania may indicate a high level of biodiversity in this species, and further investigations in terms of polymorphism, chemical and antimicrobial potential may be useful. The identified antimicrobial activity of these oils could be the way to discover new natural preservation modalities with possible uses in the food and cosmetics industry, replacing synthetic preservatives, compounds that present a higher degree of toxicity to humans and animals.

## Figures and Tables

**Table 1:** The yield and color of the EOs isolated from the aerial part of *Thymus* populations collected from five areas in Banat.

Population		EO Abbreviation	Location	EO Yield (%)	EO Color
*T. odoratissimus* Mill.	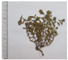	TgS	Silagiu	0.620 ± 0.012	Brown
*T. pulegioides* L.	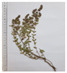	TpP	Prigor	0.460 ± 0.033	Brown–yellow
*T. pulegioides* L.	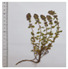	TpC	Carasova	0.440 ± 0.021	Yellow
*T. pulegioides* L.	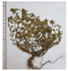	TpB	Bazias	0.490 ± 0.017	Pale-yellow
*T. vulgaris* L.	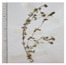	TvL	Lovrin	0.810 ± 0.042	Brown–yellow

**Table 2:** Chemical composition of the EOs isolated from aerial part of the *Thymus* population collected from five areas in Banat: *T. odoratissimus* (TgS); *T. pulegioides* (TpP); *T. pulegioides* (TpC); *T. pulegioides* (TpB); *T. vulgaris* (TvL).

Nr Crt	Ri ^a^	Ri ^b^	Compound	Classes	TgS	TpP	TpC	TpB	TvL
1	930	930	α-Thujene	MH	1.42	1.12	1	0.5	0.63
2	939	939	α-Pinene *	MH	0.67	0.59	0.5	0.25	0.5
3	954	954	Camphene	MH	0.38	0.36	0.42	0.21	
4	975	975	Sabinene *	MH	0.06	0.1	0.09	0.04	
5	979	983	β-Pinene *	MH	1.63	1.03	1.01	0.48	0.52
6	990	987	β-Myrcene *	MH			0.08		
7	1002	993	α-Phellandrene *	MH	0.31	0.21	0.16		
8	1011	1002	3-Carene *	MH	0.07	0.06	0.04		
9	1012	1010	4-Carene	MH	1.86	1.6	1.3	0.57	
10	1020	1019	D-Limonene *	MH				0.23	
11	1023	1020	*m*-Cymene	MH			8.45	6.3	
12	1024	1021	*p*-Cymene	MH	8.55	15.12	0.43		10.94
13	1037	1027	*cis*-β-Ocimene *	MH	0.22	0.99			
14	1050	1038	*trans*-β-Ocimene *	MH			0.06		
15	1059	1069	γ-Terpinene	MH	8.96	5.14	7.47	1.68	8.5
16	1070	1085	*cis*-Sabinene hydrate	MH	1.69	0.55	0.49	0.2	1.27
17	1088	1107	α-Terpinolene	MH	0.1	0.16			1.04
18	1299	1295	Carvacrol *	MO	7.94	5.71	25.43	15.92	2.58
19	1235	1238	Thymyl methyl ether	MO	6.34	5.88	2.05	1.6	5.27
20	1244	1250	Carvacrol methyl ether	MO	6.67	5.73	5.54	3.81	5.24
21	1290	1290	Thymol	MO	30.82	33.81	13.93	17.14	40.85
22	1096	1121	Linalool	MO	2.84		0.77	2.27	
23	1146	1140	Camphor	MO			0.08		
24	1169	1168	endo-Borneol	MO	0.62	0.55	0.86	0.47	0.7
25	1160	1170	Isoborneol	MO			0.55		
26	1177	1171	Terpinen-4-ol	MO	0.68	0.79		0.38	
27	1188	1190	α-Terpineol	MO		6.29			
28	1252	1259	*cis*-Geraniol	MO		1.88	13.12	28.35	
29	1257	1263	Linalyl acetate	MO					0.72
30	1285	1287	Borneol acetate	MO		0.56			
31	1381	1390	*cis*-Geranyl acetate	MO		0.72	3.33	5.7	
32	1271	1280	Lavandulol acetate	MO			1.22		
33	1422	1423	Lavandulyl isobutyrate	MO				0.13	
34	1591	1603	(R)-Lavandulyl (R)-2-methylbutanoate	MO				0.12	
35			Dihydro-1,8-cineole	MO	0.49	0.41	0.25		
36	980	984	3-Octanol	others		0.12			0.66
37	963	963	3-Octanone	others					2.6
38			1-Methyl-4-(methylethyl)-(E)-2-cyclohexenol	others	1.95				
39			4,8,8-Trimethyl-2-methylene-4-vinylbicyclo[5.2.0] nonane	others	2.39				
40			Unidentified	others					0.3
41			Methyl-3-methylenetricyclo[4.4.0.02,7] decane	others	0.43				
42			Unidentified	others		0.22			
43			Unidentified	others			0.33	0.37	
44	977	977	1-Octen-3-ol	others	0.52	0.4	0.23	0.23	0.84
45	1110	1123	3-Octanol acetate	others	0.21				
46			*cis*-4,11,11-Trimethyl-8-methylenebicyclo(7.2.0)undeca-4-ene (*cis*-caryophyllene)	SH	6.51		3.45	3.68	
47			Isoledene	SH	1.46		0.23	0.29	
48			α-Muurolene	SH	0.6		0.1		
49			β-Bisabolene	SH					7.54
50			γ-Cadinene	SH					0.17
51	1408	1410	Isocaryophyllene	SH			5.22	6.83	
52	1419	1420	Caryophyllene *	SH		4.19			5.98
53	1376	1380	α-Copaene	SH	0.9	0.4			
54	1454	1456	Humulene	SH		0.24	0.37	0.53	
55	1479	1483	gamma-Muurolene	SH	0.83	0.14	0.05		
56	1481	1485	Germacrene D	SH	1.88	0.18	1.05	1.25	2.24
57	1441	1442	Aromadendrene	SH		4.14			
58	1432	1435	β-copaene	SH					0.61
59	1583	1584	Caryophyllene oxide	SO			0.34		
60	1640	1638	Cadinol	SO				0.07	
TOTAL					100	99.39	100	99.6	99.7
**Total of Major Compounds**					**TgS**	**TpP**	**TpC**	**TpB**	**TvL**
					**Area (%)**				
Monoterpene hydrocarbonates (MH)					25.92	27.03	21.5	10.46	23.4
Monoterpene oxygenate (MO)					56.4	62.33	67.13	75.89	55.36
Total Monoterpene					82.32	89.36	88.63	86.35	78.76
Sesquiterpene hydrocarbonates (SH)					12.18	9.29	10.47	12.58	16.54
Sesquiterpene oxygenate (SO)					0	0	0.34	0.07	0
Total Sesquiterpene					12.18	9.29	10.81	12.65	16.54
Others					5.5	0.74	0.56	0.6	4.4
TOTAL					100	99.39	100	99.6	99.7

RI ^a^: retention indices were calculated in this study and were determined relative to the retention times (t_R_) values of *n*-alkanes (C10–C35); RI ^b^: retention indices from the literature. The names of the compounds identified also by comparison with standards are indicated with an asterisk (*).

**Table 3:** The optical density (OD) mean values of EOs at 540 nm.

%	*S. pyogenes*	*S. aureus*	*S. flexneri*	*P. aeruginosa*	*E. coli*	*S. typhimurium*	*H. influenza*	*C. parapsilopsis*	*C. albicans*
TgS16	0.167 ± 0.006 ^a^	0.197 ± 0.008 ^a^	0.199 ± 0.006 ^a^	0.572 ± 0.004 ^a^	0.166 ± 0.001 ^a^	0.171 ± 0.002 ^a^	0.130 ± 0.001 ^a^	0.201 ± 0.001 ^a^	0.218 ± 0.005 ^a^
TgS8	0.188 ± 0.004 ^b^	0.222 ± 0.001 ^b^	0.221 ± 0.003 ^b^	0.589 ± 0.002 ^b^	0.190 ± 0.007 ^b^	0.185 ± 0.002 ^b^	0.152 ± 0.002 ^b^	0.224 ± 0.005 ^b^	0.220 ± 0.003 ^a^
TgS4	0.192 ± 0.003 ^b^	0.244 ± 0.004 ^c^	0.221 ± 0.002 ^b^	0.594 ± 0.003 ^c^	0.209 ± 0.004 ^c^	0.193 ± 0.003 ^c^	0.202 ± 0.003 ^c^	0.241 ± 0.002 ^c^	0.222 ± 0.004 ^a^
TgS2	0.215 ± 0.002 ^c^	0.279 ± 0.002 ^d^	0.285 ± 0.001 ^c^	0.636 ± 0.012 ^d^	0.238 ± 0.006 ^d^	0.237 ± 0.013 ^d^	0.272 ± 0.003 ^d^	0.294 ± 0.005 ^d^	0.264 ± 0.005 ^b^
TpP16	0.163 ± 0.005 ^a^	0.218 ± 0.002 ^e^	0.210 ± 0.002 ^d^	0.634 ± 0.009 ^d^	0.191 ± 0.001 ^b^	0.200 ± 0.001 ^e^	0.202 ± 0.001 ^c^	0.217 ± 0.001 ^b^	0.240 ± 0.003 ^c^
TpP8	0.219 ± 0.004 ^c^	0.259 ± 0.002 ^f^	0.215 ± 0.002 ^e^	0.744 ± 0.013 ^e^	0.280 ± 0.001 ^e^	0.248 ± 0.003 ^d^	0.264 ± 0.002 ^e^	0.255 ± 0.005 ^e^	0.260 ± 0.003 ^d^
TpP4	0.289 ± 0.003 ^d^	0.361 ± 0.001 ^g^	0.348 ± 0.012 ^f^	1.144 ± 0.047 ^f^	0.328 ± 0.001 ^e^	0.315 ± 0.004 ^f^	0.296 ± 0.044 ^f,h^	0.334 ± 0.002 ^f^	0.277 ± 0.002 ^e^
TpP2	0.321 ± 0.001 ^e^	0.385 ± 0.007 ^h^	0.391 ± 0.009 ^g^	1.169 ± 0.020 ^f,g^	0.456 ± 0.008 ^e^	0.385 ± 0.008 ^g^	0.428 ± 0.004 ^g^	0.437 ± 0.004 ^g^	0.358 ± 0.001 ^f^
TpC16	0.159 ± 0.003 ^a^	0.158 ± 0.001 ^e^	0.161 ± 0.001 ^h^	1.132 ± 0.055 ^f^	0.167 ± 0.005 ^a^	0.164 ± 0.001 ^h^	0.201 ± 0.003 ^c^	0.165 ± 0.003 ^h^	0.155 ± 0.003 ^g^
TpC8	0.194 ± 0.001 ^b^	0.191 ± 0.004 ^a^	0.189 ± 0.001 ^i^	1.255 ± 0.022 ^h,g^	0.193 ± 0.002 ^b^	0.193 ± 0.002 ^c^	0.235 ± 0.003 ^i^	0.180 ± 0.001 ^i^	0.162 ± 0.002 ^h,j^
TpC4	0.204 ± 0.002 ^f^	0.218 ± 0.001 ^e^	0.192 ± 0.002 ^i^	1.375 ± 0.066 ^i^	0.202 ± 0.004 ^b^	0.202 ± 0.001 ^e^	0.240 ± 0.002 ^i^	0.205 ± 0.003 ^j^	0.218 ± 0.004 ^i^
TpC2	0.204 ± 0.002 ^f^	0.244 ± 0.003 ^c^	0.223 ± 0.002 ^j^	1.364 ± 0.032 ^i^	0.226 ± 0.009 ^d,f^	0.227 ± 0.011 ^d^	0.269 ± 0.012 ^e,h^	0.255 ± 0.010 ^e^	0.234 ± 0.002 ^k^
TpB16	0.149 ± 0.002 ^h^	0.654 ± 0.020 ^i^	0.142 ± 0.001 ^k^	0.970 ± 0.024 ^j^	0.124 ± 0.003 ^g^	0.133 ± 0.002 ^i^	0.180 ± 0.002 ^j^	0.149 ± 0.001 ^k^	0.137 ± 0.002 ^l^
TpB8	0.161 ± 0.002 ^a^	0.699 ± 0.019 ^j^	0.145 ± 0.005 ^k^	1.200 ± 0.025 ^g^	0.146 ± 0.001 ^h^	0.140 ± 0.001 ^j^	0.223 ± 0.002 ^k^	0.141 ± 0.002 ^k^	0.136 ± 0.003 ^l^
TpB4	0.162 ± 0.001 ^a^	0.754 ± 0.008 ^k^	0.166 ± 0.001 ^l^	1.365 ± 0.090 ^i^	0.178 ± 0.004 ^b^	0.150 ± 0.002 ^k^	0.446 ± 0.008 ^k^	0.177 ± 0.003 ^l^	0.136 ± 0.005 ^l^
TpB2	0.167 ± 0.003 ^a,g^	0.778 ± 0.002 ^l^	0.171 ± 0.021 ^l^	1.507 ± 0.019 ^k^	0.187 ± 0.003 ^b^	0.172 ± 0.003 ^l^	0.380 ± 0.003 ^l^	0.635 ± 0.027 ^m^	0.156 ± 0.009 ^g,j^
TvL16	0.166 ± 0.002 ^a,g^	0.179 ± 0.001 ^m^	0.181 ± 0.002 ^m^	1.393 ± 0.050 ^i^	0.184 ± 0.007 ^b^	0.183 ± 0.000 ^b^	0.202 ± 0.002 ^c^	0.185 ± 0.004 ^n^	0.157 ± 0.004 ^g,j^
TvL8	0.227 ± 0.002 ^c^	0.210 ± 0.001 ^n^	0.206 ± 0.002 ^a^	1.219 ± 0.038 ^g^	0.220 ± 0.006 ^c,f^	0.211 ± 0.001 ^m^	0.195 ± 0.000 ^m^	0.209 ± 0.003 ^o^	0.167 ± 0.002 ^h^
TvL4	0.235 ± 0.002 ^f^	0.463 ± 0.014 ^o^	0.213 ± 0.005 ^e^	0.903 ± 0.018 ^l^	0.242 ± 0.002 ^d,i^	0.214 ± 0.003 ^m^	0.203 ± 0.002 ^c^	0.250 ± 0.001 ^e^	0.237 ± 0.002 ^m^
TvL2	0.234 ± 0.004 ^f^	0.462 ± 0.003 ^o^	0.260 ± 0.009 ^n^	0.828 ± 0.002 ^m^	0.267 ± 0.022 ^i^	0.221 ± 0.005 ^d^	0.255 ± 0.001 ^j,h^	0.282 ± 0.003 ^p^	0.268 ± 0.002 ^n^
M	0.475 ± 0.005 ^i^	0.438 ± 0.031 ^o^	0.798 ± 0.050 ^o^	1.284 ± 0.005 ^g^	0.462 ± 0.021 ^e^	0.574 ± 0.021 ^m^	0.337 ± 0.002 ^n^	0.863 ± 0.005 ^q^	0.377 ± 0.004 ^o^

The values are expressed as mean values ± standard deviations of all measurements. Different letters in the columns indicates significant differences (*p* < 0.05) between values according to the *t*-test.

**Table 4:** MIC (µL EO/100 µL) for TgS, TpP, TpC, TpB and TvL.

	*S. pyogenes* (ATCC 19615)	*S. aureus* (ATCC 25923)	*S. flexneri* (ATCC 120022)	*P. aeruginosa* (ATCC 27853)	*E. coli* (ATCC 25922)	*S. typhimurium* (ATCC 140028)	*H. influenzae* type B (ATCC 100211)	*C. parapsilopsis* (ATCC 220019)	*C. albicans* (ATCC 100231)
TgS	16	16	16	16	16	16	16	16	16
TgS	8	8	8	8	8	8	8	8	8
TgS	4	4	4	4	4	4	4	4	4
TgS	2	2	2	2	2	2	2	2	2
TpP	16	16	16	16	16	16	16	16	16
TpP	8	8	8	8	8	8	8	8	8
TpP	4	4	4	4	4	4	4	4	4
TpP	2	2	2	2	2	2	2	2	2
TpC	16	16	16	16	16	16	16	16	16
TpC	8	8	8	8	8	8	8	8	8
TpC	4	4	4	4	4	4	4	4	4
TpC	2	2	2	2	2	2	2	2	2
TpB	16	16	16	16	16	16	16	16	16
TpB	8	8	8	8	8	8	8	8	8
TpB	4	4	4	4	4	4	4	4	4
TpB	2	2	2	2	2	2	2	2	2
TvL	16	16	16	16	16	16	16	16	16
TvL	8	8	8	8	8	8	8	8	8
TvL	4	4	4	4	4	4	4	4	4
TvL	2	2	2	2	2	2	2	2	2

The samples that had a strain-boosting effect, maintained with an increase in concentration, are marked in dark grey color. The light grey color represents the samples that had a strain-boosting effect that decreased with the concentration. The white color highlights the samples where the MIC was determined, with the MIC value highlighted. The effect was maintained together with an increase in concentration.

**Table 5:** Species of the genus *Thymus* analyzed and their location.

Code	Analyzed Species	Location	Voucher Specimen Number	County	GPS Coordinates (Decimal Degree)		
					Altitude (m)	Latitude	Longitude
TgS	*Thymus odoratissimus* Mill.	Silagiu	VSNH.BUASTM: 1931	Timis	192	45.60703	21.60335
TgP	*Thymus pulegioides* L.	Prigor	VSNH.BUASTM: 1934	Caras-Severin	347	44.932547	22.115320
TgC	*Thymus pulegioides* L.	Carasova	VSNH.BUASTM: 1932	Caras-Severin	568	45.152790	21.878892
TgB	*Thymus pulegioides* L.	Bazias	VSNH.BUASTM: 1933	Caras-Severin	120	44.823742	21.387344
TvL	*Thymus vulgaris* L.	Lovrin	VSNH.BUASTM: 1927	Timis	91	45.975270	20.789621

## Data Availability

Data supporting reported results can be found at Interdisciplinary Research Platform within the Banat University of Agricultural Sciences and Veterinary Medicine “King Mihai I of Romania”, Timisoara, Romania and at Institute for Research, Development and Innovation in Technical and Natural Sciences, Aurel Vlaicu University, Arad, Romania.

## Supplementary Materials

The following are available online at https://www.mdpi.com/article/10.3390/plants10091833/s1, Supplementary Materials Figure S1: The individual chromatograms of *Thymus* EOs, Supplementary materials Table S2: The analysis of correlation between the antimicrobial effect of *Thymus* species against the analyzed strains and the chemical composition.

## References

[1] Rustaiee A., Sefidkon F., Tabatabaei S.M.F., Omidbaigi R., Mirahmadi S.F., Shayganfar A. (2011). Chemical Polymorphism of Essential Oils from Five Populations of *Thymus daenensis* Celak. subsp. *daenensis* Endemic to Iran. J. Essent. Oil Res..

[2] Pluhár Z., Kocsis M., Kuczmog A., Csete S., Simkó H., Sárosi S., Molnar P., Horváth G. (2012). Essential oil composition and preliminary molecular study of four Hungarian *Thymus* species. Acta Biol. Hung..

[3] Morales R., Stahl-Biskup E., Saez F. (2002). The history, botany and taxonomy of the genus *Thymus*. Thyme: The Genus Thymus.

[4] Ložienė K., Venskutonis P. (2005). Influence of environmental and genetic factors on the stability of essential oil composition of *Thymus pulegioides*. Biochem. Syst. Ecol..

[5] Taghouti M., Martins-Gomes C., Félix L.M., Schäfer J., Santos J.A., Bunzel M., Nunes F.M., Silva A.M. (2020). Polyphenol composition and biological activity of *Thymus citriodorus* and *Thymus vulgaris*: Comparison with endemic Iberian *Thymus species*. Food Chem..

[6] Gușuleac M., Săvulescu T.R., Redactore Tomi Nyarady E.I., Beldie A., Bioa A., Grințescu I., Gușuleac M., Moraiu I., Nyarady A., Nyarady E.I., Paucă A. (1961). *Thymus* L. Flora Romaniae.

[7] Sârbu I., Ștefan N., Oprea A. (2013). Plante Vasculare din România. Determinator Ilustrat de Teren.

[8] The EuroPlusMedPlantbase. https://ww2.bgbm.org/EuroPlusMed/query.asp.

[9] Ramchoun M., Khouya T., Harnafi H., Amrani S., Alem C., Benlyas M., Chadli F.K., Nazih H., Nguyen P., Ouguerram K. (2020). Effect of Aqueous Extract and Polyphenol Fraction Derived from *Thymus atlanticus* Leaves on Acute Hyperlipidemia in the Syrian Golden Hamsters. Evid.-Based Complement. Altern. Med..

[10] Hoven R.V.D., Zappe H., Zitterl-Eglseer K., Jugl M., Franz C. (2003). Study of the effect of Bronchipret on the lung function of five Austrian saddle horses suffering recurrent airway obstruction (heaves). Veter. Rec..

[11] Nascimento G.G.F., Locatelli J., Freitas P.C., Silva G.L. (2000). Antibacterial activity of plant extracts and phytochemicals on antibiotic-resistant bacteria. Braz. J. Microbiol..

[12] Ciobotaru V.G.G., Pavel I.Z., Borcan F., Moaca A., Danciu C., Diaconeasa Z., Imbrea I., Vlad D., Dumitrascu V., Pop G. (2019). Toxicological Evaluation of Some Essential Oils Obtained from Selected Romania Lamiaceae Species in Complex with Hydroxypropyl—Gamma-cyclodextrin. Rev. Chim..

[13] Alexa E., Sumalan R.M., Danciu C., Obistioiu D., Negrea M., Poiana M.-A., Rus C., Radulov I., Pop G., Dehelean C. (2018). Synergistic Antifungal, Allelopatic and Anti-Proliferative Potential of *Salvia officinalis* L., and *Thymus vulgaris* L. Essential Oils. Molecules.

[14] Granger R., Passet J. (1973). *Thymus vulgaris* spontane de France: Races chimiques et chemotaxonomie. Phytochemistry.

[15] Semeniuc C.A., Socaciu M.-I., Socaci S.A., Mureșan V., Nagy M., Rotar A.M. (2018). Chemometric Comparison and Classification of Some Essential Oils Extracted from Plants Belonging to Apiaceae and Lamiaceae Families Based on Their Chemical Composition and Biological Activities. Molecules.

[16] Pinto E., Gonçalves M.J., Hrimpeng K., Pinto J., Vaz S., Vale-Silva L.A., Cavaleiro C., Salgueiro L. (2013). Antifungal activity of the essential oil of Thymus villosus subsp. lusitanicus against Candida, Cryptococcus, Aspergillus and dermatophyte species. Ind. Crop. Prod..

[17] Lawrence B.M., Tucker A.O., Stahl-Biskup E., Saez F. (2002). The genus *Thymus* as a source of commercial products. Thyme: The Genus Thymus.

[18] Hadian J., Bigdeloo M., Nazeri V., Khadivi-Khub A. (2014). Assessment of genetic and chemical variability in Thymus caramanicus. Mol. Biol. Rep..

[19] Tohidi B., Rahimmalek M., Arzani A., Sabzalian M.R. (2020). Thymol, carvacrol, and antioxidant accumulation in *Thymus* species in response to different light spectra emitted by light-emitting diodes. Food Chem..

[20] Ghasemi P., Barani M., Hamedi B., Ataei K., Karimi A. (2013). Environment effect on diversity in quality and quantity of essential oil of different wild populations of Kerman thyme. Genetika.

[21] Vladimir-Knežević S., Blažeković B., Kindl M., Vladić J., Lower-Nedza A.D., Brantner A.H. (2014). Acetylcholinesterase Inhibitory, Antioxidant and Phytochemical Properties of Selected Medicinal Plants of the Lamiaceae Family. Molecules.

[22] Cocan I., Alexa E., Danciu C., Radulov I., Galuscan A., Obistioiu D., Morvay A.A., Sumalan R.M., Poiana M.-A., Pop G. (2017). Phytochemical screening and biological activity of Lamiaceae family plant extracts. Exp. Ther. Med..

[23] Nieto G. (2017). Biological Activities of Three Essential Oils of the Lamiaceae Family. Medicines.

[24] Karpiński T.M. (2020). Essential Oils of Lamiaceae Family Plants as Antifungals. Biomolecules.

[25] Iseppi R., Tardugno R., Brighenti V., Benvenuti S., Sabia C., Pellati F., Messi P. (2020). Phytochemical Composition and In Vitro Antimicrobial Activity of Essential Oils from the *Lamiaceae* Family against *Streptococcus agalactiae* and *Candida albicans* Biofilms. Antibiotics.

[26] Ložienė K., Vaičiulytė V., Maždžierienė R. (2021). Influence of meteorological conditions on essential oil composition in geraniol-bearing *Thymus pulegioides* and *Thymus* hybrid. Acta Physiol. Plant..

[27] Imelouane B., Amhamdi H., Wathelet J.P., Ankit M., Khedid K., El Bachiri A. (2009). Chemical composition and antimicrobial activity of essential oil of thyme (*Thymus vulgaris*) from Eastern Morocco. Int. J. Agric. Biol..

[28] Golkar P., Mosavat N., Jalali S.A.H. (2020). Essential oils, chemical constituents, antioxidant, antibacterial and in vitro cytotoxic activity of different Thymus species and Zataria multiflora collected from Iran. S. Afr. J. Bot..

[29] Pavel M., Ristić M., Stević T. (2010). Essential oils of Thymus pulegioides and Thymus glabrescens from Romania: Chemical composition and antimicrobial activity. J. Serb. Chem. Soc..

[30] Mamadalieva N.Z., Akramov D.K., Ovidi E., Tiezzi A., Nahar L., Azimova S.S., Sarker S.D. (2017). Aromatic Medicinal Plants of the Lamiaceae Family from Uzbekistan: Ethnopharmacology, Essential Oils Composition, and Biological Activities. Medicines.

[31] Boros B., Jakabová S., Dornyei A., Horváth G., Pluhár Z., Kilar F., Felinger A. (2010). Determination of polyphenolic compounds by liquid chromatography–mass spectrometry in Thymus species. J. Chromatogr. A.

[32] Borugă O., Jianu C., Mişcă C., Goleţ I., Gruia A.T., Horhat F.G. (2014). *Thymus vulgaris* essential oil: Chemical composition nd antimicrobial activity. J. Med. Life.

[33] Isopencu G., Ferdes M. (2012). Aspects regarding the influence of concentration of compoments with antifungal activity from some essential oils. Rev. Chim..

[34] Grigore A., Mihul A., Paraschiv I., Nita S., Christof R., Iuksel R., Ichim M. (2012). Chemical analysis and antimicrobial activity of indigenous medicinal species volatile oils. Rom. Biotechnol. Lett..

[35] Varga E., Bardocz A., Belak A., Maraz A., Boros B., Felinger A., Boszormenyi A., Horvath G. (2015). Antimicrobial activity and chemical composition of thyme essential oils and the polyphenolic content of different Thymus extract. Farmacia.

[36] Lorenzo J.M., Munekata P.E.S., Dominguez R., Pateiro M., Saraiva J.A., Franco D. (2018). Main Groups of Microorganisms of Relevance for Food Safety and Stability. Innov. Technol. Food Preserv..

[37] Park S.-N., Lim Y.K., Freire M., Cho E., Jin D., Kook J.-K. (2012). Antimicrobial effect of linalool and α-terpineol against periodontopathic and cariogenic bacteria. Anaerobe.

[38] Rehab M.A.E.-B., Zeinab S.H. (2016). Eugenol and linalool: Comparison of their antibacterial and antifungal activities. Afr. J. Microbiol. Res..

[39] Ložienė K., Venskutonis P.R., Šipailienė A., Labokas J. (2007). Radical scavenging and antibacterial properties of the extracts from different *Thymus pulegioides* L. chemotypes. Food Chem..

[40] Mockute D., Bernotiene G. (2003). Five Chemotypes of the Essential Oils of *Thymus pulegioides* L. Growing Wild in Lithuania. J. Essent. Oil Bear. Plants.

[41] De Martino L., Bruno M., Formisano C., De Feo V., Napolitano F., Rosselli S., Senatore F. (2009). Chemical Composition and Antimicrobial Activity of the Essential Oils from Two Species of Thymus Growing Wild in Southern Italy. Molecules.

[42] Duman A.D., Telci I., Dayisoylu K.S., Digrak M., Demirtas I., Alma M.H. (2010). Evaluation of Bioactivity of Linalool-rich Essential Oils from Ocimum basilucum and Coriandrum sativum Varieties. Nat. Prod. Commun..

[43] Afonso A.F., Pereira O.R., Válega M., Silva A.M.S., Cardoso S.M. (2018). Metabolites and Biological Activities of Thymus zygis, *Thymus pulegioides*, and Thymus fragrantissimus Grown under Organic Cultivation. Molecules.

[44] Vaičiulytė V., Ložienė K., Švedienė J., Raudonienė V., Paškevičius A. (2021). α-Terpinyl Acetate: Occurrence in Essential Oils Bearing *Thymus pulegioides*, Phytotoxicity, and Antimicrobial Effects. Molecules.

[45] Niculae M., Hanganu D., Oniga I., Benedec D., Ielciu I., Giupana R., Sandru C.D., Ciocârlan N., Spinu M. (2019). Phytochemical Profile and Antimicrobial Potential of Extracts Obtained from Thymus marschallianus Willd. Molecules.

[46] Mancini E., Senatore F., Del Monte D., De Martino L., Grulova D., Scognamiglio M., Snoussi M., De Feo V. (2015). Studies on Chemical Composition, Antimicrobial and Antioxidant Activities of Five *Thymus vulgaris* L. Essential Oils. Molecules.

[47] Verma R.S., Padalia R.C., Saikia D., Chauhan A., Krishna V., Sundaresan V. (2016). Chemical Composition and Antimicrobial Activity of the Essential Oils Isolated from the Herbage and Aqueous Distillates of two *Thymus* Species. J. Essent. Oil Bear. Plants.

[48] Al-Shuneigat J., Al-Sarayreh S., Al-Saraireh Y., Al-Qudah M., Al-Tarawneh I., Al Bataineh E. (2014). Effects of wild *Thymus vulgaris* essential oil on clinical isolates biofilm-forming bacteria. IOSR J. Dent. Med Sci..

[49] Soković M.D., Vukojevic J., Marin P.D., Brkić D.D., Vajs V., Van Griensven L.J.L.D. (2009). Chemical Composition of Essential Oilsof Thymus and Mentha Speciesand Their Antifungal Activities. Molecules.

[50] De Carvalho R.J., de Souza G.T., Honório V.G., de Sousa J.P., da Conceição M.L., Maganani M., de Souza E.L. (2015). Comparative inhibitory effects of *Thymus vulgaris* L. essential oil against Staphylococcus aureus, Listeria monocytogenes and mesophilic starter co-culture in cheese-mimicking models. Food Microbiol..

[51] Ahmad A., Van Vuuren S., Viljoen A. (2014). Unravelling the Complex Antimicrobial Interactions of Essential Oils—The Case of *Thymus vulgaris* (*Thyme*). Molecules.

[52] Galovičová L., Borotová P., Valková V., Vukovic N., Vukic M., Terentjeva M., Štefániková J., Ďúranová H., Kowalczewski P., Kačániová M. (2021). *Thymus serpyllum* Essential Oil and Its Biological Activity as a Modern Food Preserver. Plants.

[53] Guimarães A.C., Meireles L.M., Lemos M.F., Guimarães M.C.C., Endringer D.C., Fronza M., Scherer R. (2019). Antibacterial Activity of Terpenes and Terpenoids Present in Essential Oils. Molecules.

[54] Marchese A., Arciola C.R., Barbieri R., Silva A.S., Nabavi S.M., Sokeng A.J.T., Izadi M., Jafari N.J., Suntar I., Daglia M. (2017). Update on Monoterpenes as Antimicrobial Agents: A Particular Focus on p-Cymene. Materials.

[55] Togashi N., Inoue Y., Hamashima H., Takano A. (2008). Effects of Two Terpene Alcohols on the Antibacterial Activity and the Mode of Action of Farnesol against Staphylococcus aureus. Molecules.

[56] Lorenzi V., Muselli A., Bernardini A.F., Berti L., Pagès J.-M., Amaral L., Bolla J.-M. (2009). Geraniol Restores Antibiotic Activities against Multidrug-Resistant Isolates from Gram-Negative Species. Antimicrob. Agents Chemother..

[57] Hrytsyna M.R., Kryvtsova M.V., Salamon I., Skybitska M.I. (2020). Promising ex situ essential oil from *Thymus camphoratus* (Lamiaceae). Regul. Mech. Biosyst..

[58] Kryvtsova M.V., Salamon I., Koscova J., Bucko D., Spivak M. (2019). Antimicrobial, antibiofilm and biochemichal properties of *Thymus vulgaris* essential oil against clinical isolates of opportunistic infections. Biosyst. Divers..

[59] Adams R.P. (2007). Identification of Essential Oil Components by Gas Chromatography/Mass Spectrometry.

[60] Obistioiu D., Cocan I., Tîrziu E., Herman V., Negrea M., Cucerzan A., Neacsu A.-G., Cozma A., Nichita I., Hulea A. (2021). Phytochemical Profile and Microbiological Activity of Some Plants Belonging to the Fabaceae Family. Antibiotics.

[61] Alexa V.T., Galuscan A., Popescu I., Tirziu E., Obistioiu D., Floare A.D., Perdiou A., Jumanca D. (2019). Synergistic/Antagonistic Potential of Natural Preparations Based on Essential Oils Against Streptococcus mutans from the Oral Cavity. Molecules.

[62] Costa L.C.B., Pinto J.E.B.P., Bertolucci S.K.V., Costa J.C.D.B., Alves P.B., Niculau E.D.S. (2015). In vitroantifungal activity of Ocimum selloi essential oil and methylchavicol against phytopathogenic fungi. Rev. Cienc. Agron..

