# Supplementary material for: Antimicrobial Potential and Phytochemical Profile of Wild and Cultivated Populations of Thyme (Thymus sp.) Growing in Western Romania
Source: Plants (Basel). 2021 Sep 3;10(9):1833. doi: 10.3390/plants10091833 (PMC8465029; doi:10.3390/plants10091833)
Supplement: Supplementary file 1 [file plants-10-01833-s001.zip › Supplementary Materials S1 The individual chromatograms of Thymus EOs.pdf]

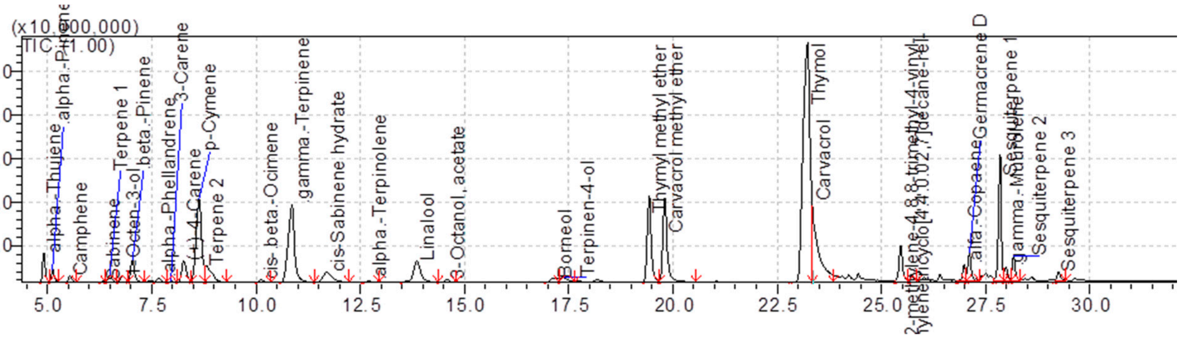
(x10,000,000)
TIC (1.00)
alpha.-Thujene
Camphene
Sabinene
Terpene 1
1-Octen-3-ol
beta.-Pinene
alpha.-Phellandrene
3-Carene
(+)-4-Carene
p-Cymene
Terpene 2
cis-.beta.-Ocimene
gamma.-Terpinene
cis-Sabinene hydrate
alpha.-Terpinolene
Linalool
3-Octanol, acetate
Borneol
Terpinen-4-ol
Thymol methyl ether
Carvacrol methyl ether
Carvacrol
Thymol
alfa.-Copaene
Germacrene D
gamma.-Muurolene
Sesquiterpene 1
Sesquiterpene 2
Sesquiterpene 3
5.0
7.5
10.0
12.5
15.0
17.5
20.0
22.5
25.0
27.5
30.0

(**a**)

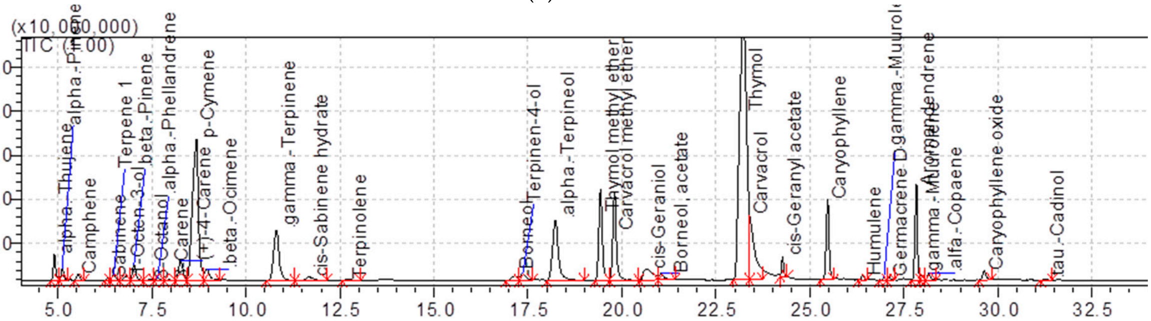
(x10,000,000)
TIC (1.00)
alpha.-Thujene
Camphene
Sabinene
Terpene 1
1-Octen-3-ol
beta.-Pinene
3-Octanol
Carene
alpha.-Phellandrene
(+)-4-Carene
p-Cymene
beta.-Ocimene
gamma.-Terpinene
cis-Sabinene hydrate
Terpinolene
Borneol
Terpinen-4-ol
alpha.-Terpineol
Thymol methyl ether
Carvacrol methyl ether
cis-Geraniol
Borneol, acetate
Carvacrol
Thymol
cis-Geranyl acetate
Caryophyllene
Humulene
Germacrene D
gamma.-Muurolene
alfa.-Copaene
Caryophyllene oxide
tau.-Cadinol
5.0
7.5
10.0
12.5
15.0
17.5
20.0
22.5
25.0
27.5
30.0
32.5

(**b**)

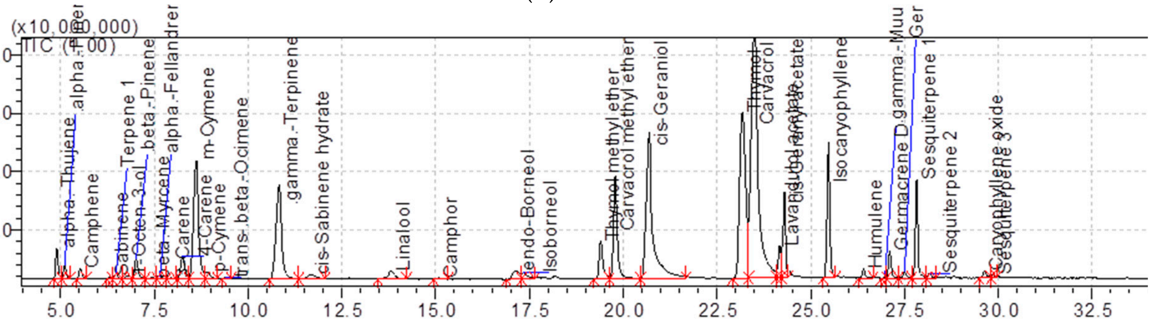
(x10,000,000)
TIC (1.00)
alpha.-Thujene
Camphene
Sabinene
Terpene 1
1-Octen-3-ol
beta.-Pinene
beta.-Myrcene
Carene
alpha.-Fellandrene
4-Carene
p-Cymene
m-Cymene
trans-.beta.-Ocimene
gamma.-Terpinene
cis-Sabinene hydrate
Linalool
Camphor
endo-Borneol
Isoborneol
Thymol methyl ether
Carvacrol methyl ether
cis-Geraniol
Thymol
Carvacrol
Isocaryophyllene
Humulene
Germacrene D
Sesquiterpene 1
Sesquiterpene 2
5.0
7.5
10.0
12.5
15.0
17.5
20.0
22.5
25.0
27.5
30.0
32.5

(**c**)

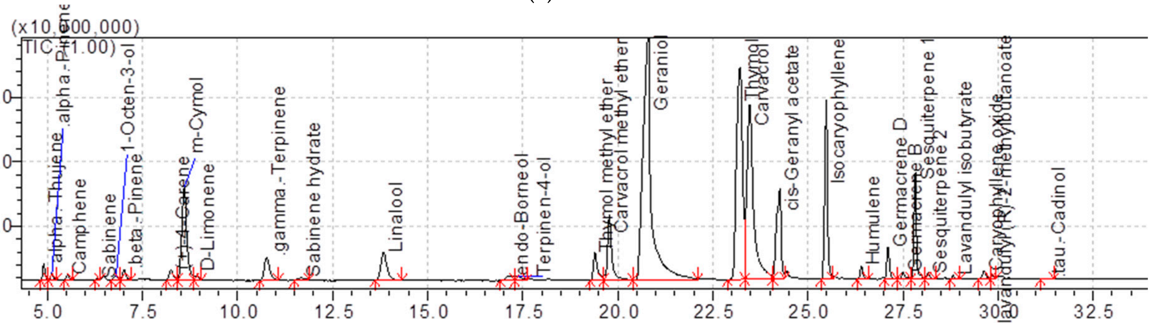
(x10,000,000)
TIC (1.00)
alpha.-Thujene
Camphene
Sabinene
1-Octen-3-ol
beta.-Pinene
(+)-4-Carene
m-Cymol
D-Limonene
gamma.-Terpinene
Sabinene hydrate
Linalool
endo-Borneol
Terpinen-4-ol
Thymol methyl ether
Carvacrol methyl ether
Geraniol
Thymol
Carvacrol
cis-Geranyl acetate
Isocaryophyllene
Humulene
Germacrene D
Germacrene B
Sesquiterpene 1
Sesquiterpene 2
Lavandulyl isobutyrate
tau.-Cadinol
5.0
7.5
10.0
12.5
15.0
17.5
20.0
22.5
25.0
27.5
30.0
32.5

(**d**)

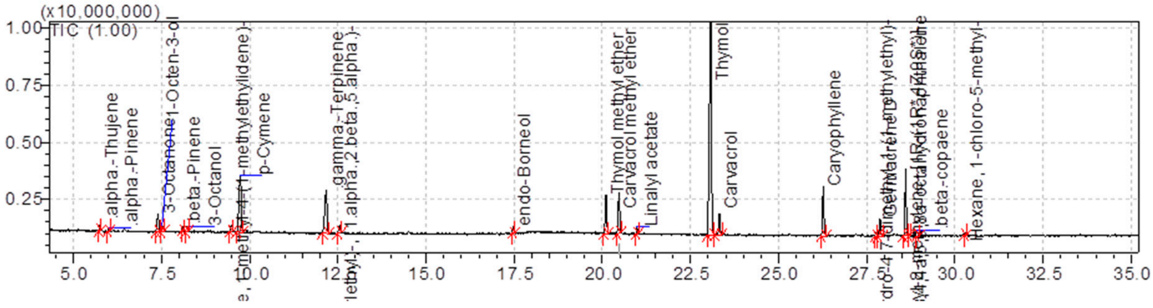

(**e**)

Supplementary Materials S1: The individual chromatograms of *Thymus* EOs

(**a**) Thymus glabrescens EO **TgS**; (**b**) Thymus pulegioides EO **TpP**; (**c**) Thymus pulegioides EO **TpC**; (**d**) Thymus pulegioides EO **TpB**; (**e**) Thymus vulgaris EO **TvL**
