# Supplementary material for: Antimicrobial Potential and Phytochemical Profile of Wild and Cultivated Populations of Thyme (Thymus sp.) Growing in Western Romania
Source: Plants (Basel). 2021 Sep 3;10(9):1833. doi: 10.3390/plants10091833 (PMC8465029; doi:10.3390/plants10091833)
Supplement: Supplementary file 1 [file plants-10-01833-s001.zip › Supplementary Materials S2.pdf]

Supplementary Materials S2. The analysis of correlation between the antimicrobial effect of *Thymus* species against the analyzed strains and the chemical composition. Pearson correlation between the chemical composition and OD values for TgS, TpP, TpC, TpB, TvL 16%

| **TgS** | *S. pyogenes* | *S. aureus* | *S. flexneri* | *P. aeruginosa* | *E. coli* | *S. typhimurium* | *H. influenzae* | *C. parapsilopsis* | *C. albicans* | Carvacrol | Thymol | p-Cymene | γ-Terpinene | Linalool |
|---|---|---|---|---|---|---|---|---|---|---|---|---|---|---|
| *S. pyogenes* | 1 | | | | | | | | | | | | | |
| *S. aureus* | -0.569 | 1 | | | | | | | | | | | | |
| *S. flexneri* | -0.569 | 1 | 1 | | | | | | | | | | | |
| *P. aeruginosa* | -0.980 | 0.721 | 0.721 | 1 | | | | | | | | | | |
| *E. coli* | -0.082 | 0.866 | 0.866 | 0.277 | 1 | | | | | | | | | |
| *S. typhimurium* | 0.807 | -0.945 | -0.945 | -0.908 | -0.655 | 1 | | | | | | | | |
| *H. influenzae* | -0.997 | 0.500 | 0.500 | 0.961 | 0.000 | -0.756 | 1 | | | | | | | |
| *C. parapsilopsis* | 0.569 | -1.000 | -1.000 | -0.721 | -0.866 | 0.945 | -0.500 | 1 | | | | | | |
| *C. albicans* | 0.249 | 0.655 | 0.655 | -0.052 | 0.945 | -0.371 | -0.327 | -0.655 | 1 | | | | | |
| Carvacrol | 0.783 | -0.957 | -0.957 | -0.890 | -0.684 | 0.999 | -0.729 | 0.957 | -0.408 | 1 | | | | |
| Thymol | 0.166 | -0.905 | -0.905 | -0.358 | -0.996 | 0.716 | -0.085 | 0.905 | -0.914 | 0.744 | 1 | | | |
| p-Cymene | 0.904 | -0.866 | -0.866 | -0.971 | -0.500 | 0.982 | -0.866 | 0.866 | -0.189 | 0.974 | 0.572 | 1 | | |
| γ-Terpinene | 0.852 | -0.916 | -0.916 | -0.938 | -0.592 | 0.997 | -0.806 | 0.916 | -0.296 | 0.993 | 0.658 | 0.994 | 1 | |
| Linalool | -0.528 | -0.397 | -0.397 | 0.350 | -0.803 | 0.075 | 0.596 | 0.397 | -0.954 | 0.115 | 0.749 | -0.115 | -0.005 | 1 |

| **TpP** | *S. pyogenes* | *S. aureus* | *S. flexneri* | *P. aeruginosa* | *E. coli* | *S. typhimurium* | *H. influenzae* | *C. parapsilopsis* | *C. albicans* | Carvacrol | Thymol | p-Cymene | γ-Terpinene | Geraniol |
|---|---|---|---|---|---|---|---|---|---|---|---|---|---|---|
| S. pyogenes | 1 | | | | | | | | | | | | | |
| S. aureus | -0.106 | 1 | | | | | | | | | | | | |
| S. flexneri | 0.858 | 0.419 | 1 | | | | | | | | | | | |
| P. aeruginosa | 0.000 | -0.994 | -0.513 | 1 | | | | | | | | | | |
| E. coli | -0.225 | -0.945 | -0.693 | 0.974 | 1 | | | | | | | | | |
| S. typhimurium | 0.731 | -0.756 | 0.277 | 0.682 | 0.500 | 1 | | | | | | | | |
| H. influenzae | 0.468 | -0.929 | -0.052 | 0.884 | 0.756 | 0.945 | 1 | | | | | | | |
| C. parapsilopsis | 0.731 | -0.756 | 0.277 | 0.682 | 0.500 | 1.000 | 0.945 | 1 | | | | | | |
| C. albicans | -0.589 | 0.866 | -0.091 | -0.808 | -0.655 | -0.982 | -0.990 | -0.982 | 1 | | | | | |
| Carvacrol | -0.982 | 0.291 | -0.747 | -0.187 | 0.038 | -0.846 | -0.625 | -0.846 | 0.730 | 1 | | | | |
| Thymol | -0.998 | 0.171 | -0.823 | -0.065 | 0.161 | -0.774 | -0.525 | -0.774 | 0.641 | 0.992 | 1 | | | |
| p-Cymene | 0.925 | 0.280 | 0.989 | -0.381 | -0.579 | 0.417 | 0.096 | 0.417 | -0.237 | -0.837 | -0.898 | 1 | | |
| γ-Terpinene | -0.997 | 0.189 | -0.812 | -0.084 | 0.143 | -0.786 | -0.540 | -0.786 | 0.655 | 0.995 | 1.000 | -0.890 | 1 | |
| Geraniol | 0.914 | -0.500 | 0.577 | 0.405 | 0.189 | 0.945 | 0.786 | 0.945 | -0.866 | -0.974 | -0.939 | 0.691 | -0.945 | 1 |

| **TpC** | *S. pyogenes* | *S. aureus* | *S. flexneri* | *P. aeruginosa* | *E. coli* | *S. typhimurium* | *H. influenzae* | *C. parapsilopsis* | *C. albicans* | Carvacrol | Thymol | p-Cymene | γ-Terpinene | Geraniol | Linalool |
|---|---|---|---|---|---|---|---|---|---|---|---|---|---|---|---|
| *S. pyogenes* | 1 | | | | | | | | | | | | | | |
| *S. aureus* | 0.189 | 1 | | | | | | | | | | | | | |
| *S. flexneri* | 0.655 | 0.866 | 1 | | | | | | | | | | | | |
| *P. aeruginosa* | 0.163 | -0.938 | -0.639 | 1 | | | | | | | | | | | |
| *E. coli* | 0.999 | 0.225 | 0.682 | 0.127 | 1 | | | | | | | | | | |
| *S. typhimurium* | 0.756 | -0.500 | 0.000 | 0.769 | 0.731 | 1 | | | | | | | | | |
| *H. influenzae* | 0.984 | 0.359 | 0.778 | -0.013 | 0.990 | 0.629 | 1 | | | | | | | | |
| *C. parapsilopsis* | 0.000 | 0.982 | 0.756 | -0.987 | 0.037 | -0.655 | 0.176 | 1 | | | | | | | |
| *C. albicans* | -0.327 | 0.866 | 0.500 | -0.986 | -0.292 | -0.866 | -0.156 | 0.945 | 1 | | | | | | |
| *Carvacrol* | 0.999 | 0.146 | 0.621 | 0.206 | 0.997 | 0.784 | 0.976 | -0.043 | -0.368 | 1 | | | | | |
| *Thymol* | -0.955 | 0.111 | -0.401 | -0.449 | -0.943 | -0.916 | -0.888 | 0.297 | 0.593 | -0.967 | 1 | | | | |
| *p-Cymene* | 0.655 | 0.866 | 1.000 | -0.639 | 0.682 | 0.000 | 0.778 | 0.756 | 0.500 | 0.621 | -0.401 | 1 | | | |
| γ -Terpinene | -1.000 | -0.171 | -0.640 | -0.182 | -0.998 | -0.768 | -0.981 | 0.019 | 0.345 | -1.000 | 0.960 | -0.640 | 1 | | |
| Geraniol | 0.800 | 0.741 | 0.977 | -0.462 | 0.821 | 0.212 | 0.893 | 0.600 | 0.305 | 0.773 | -0.586 | 0.977 | -0.788 | 1 | |
| Linalool | -0.990 | -0.327 | -0.756 | -0.021 | -0.994 | -0.655 | -0.999 | -0.143 | 0.189 | -0.983 | 0.903 | -0.756 | 0.987 | -0.877 | 1 |

| **TpB** | *S. pyogenes* | *S. aureus* | *S. flexneri* | *P. aeruginosa* | *E. coli* | *S. typhimurium* | *H. influenzae* | *C. parapsilopsis* | *C. albicans* | Carvacrol | Thymol | γ-Terpinene | cis-Geraniol | Linalool |
|---|---|---|---|---|---|---|---|---|---|---|---|---|---|---|
| *S. pyogenes* | 1 | | | | | | | | | | | | | |
| *S. aureus* | -0.640 | 1 | | | | | | | | | | | | |
| *S. flexneri* | -0.866 | 0.939 | 1 | | | | | | | | | | | |
| *P. aeruginosa* | -0.952 | 0.375 | 0.672 | 1 | | | | | | | | | | |
| *E. coli* | -0.866 | 0.171 | 0.500 | 0.977 | 1 | | | | | | | | | |
| *S. typhimurium* | 0.655 | 0.161 | -0.189 | -0.854 | -0.945 | 1 | | | | | | | | |
| *H. influenzae* | -0.945 | 0.857 | 0.982 | 0.800 | 0.655 | -0.371 | 1 | | | | | | | |
| *C. parapsilopsis* | 0.000 | -0.768 | -0.500 | 0.305 | 0.500 | -0.756 | -0.327 | 1 | | | | | | |
| *C. albicans* | 0.000 | 0.768 | 0.500 | -0.305 | -0.500 | 0.756 | 0.327 | -1 | 1 | | | | | |
| Carvacrol | -0.814 | 0.076 | 0.415 | 0.553 | 0.995 | -0.972 | 0.579 | 0.581 | -0.581 | 1 | | | | |
| Thymol | -0.866 | 0.171 | 0.500 | 0.477 | 1.000 | -0.945 | 0.655 | 0.500 | -0.500 | 0.995 | 1 | | | |
| γ -Terpinene | -0.610 | -0.218 | 0.132 | 0.423 | 0.924 | -0.998 | 0.317 | 0.792 | -0.792 | 0.957 | 0.924 | 1 | | |
| cis-geraniol | -0.810 | 0.069 | 0.409 | 0.351 | 0.995 | -0.973 | 0.574 | 0.586 | -0.586 | 1.000 | 0.995 | 0.959 | 1 | |
| Linalool | -0.564 | -0.273 | 0.075 | 0.589 | 0.901 | -0.993 | 0.262 | 0.826 | -0.826 | 0.939 | 0.901 | 0.998 | 0.941 | 1 |

| **TvL** | *S. pyogenes* | *S. aureus* | *S. flexneri* | *P. aeruginosa* | *E. coli* | *S. typhimurium* | *H. influenzae* | *C. parapsilopsis* | *C. albicans* | Carvacrol | Thymol | p-Cymene | γ -Terpinene |
|---|---|---|---|---|---|---|---|---|---|---|---|---|---|
| *S. pyogenes* | 1 | | | | | | | | | | | | |
| *S. aureus* | -0.189 | 1 | | | | | | | | | | | |
| *S. flexneri* | -0.189 | 1 | 1 | | | | | | | | | | |
| *P. aeruginosa* | 0.862 | 0.336 | 0.336 | 1 | | | | | | | | | |
| *E. coli* | -0.262 | 0.997 | 0.997 | 0.264 | 1 | | | | | | | | |
| *S. typhimurium* | 0.000 | 0.000 | 0.000 | 0.000 | 0.000 | 1 | | | | | | | |
| *H. influenzae* | 0.954 | 0.115 | 0.115 | 0.974 | 0.040 | 0 | 1 | | | | | | |
| *C. parapsilopsis* | 0.317 | -0.991 | -0.991 | -0.208 | -0.998 | 0 | 0.017 | 1 | | | | | |
| *C. albicans* | -0.663 | -0.610 | -0.610 | -0.951 | -0.549 | 0 | -0.857 | 0.500 | 1 | | | | |
| Carvacrol | -1.000 | 0.189 | 0.189 | -0.862 | 0.262 | 0 | -0.954 | -0.317 | 0.663 | 1 | | | |
| Thymol | -0.771 | 0.771 | 0.771 | -0.341 | 0.817 | 0 | -0.544 | -0.848 | 0.034 | 0.771 | 1 | | |
| p-Cymene | -0.011 | -0.980 | -0.980 | -0.517 | -0.962 | 0 | -0.311 | 0.945 | 0.756 | 0.011 | -0.628 | 1 | |
| γ-Terpinene | -0.032 | -0.975 | -0.975 | -0.535 | -0.956 | 0 | -0.331 | 0.938 | 0.770 | 0.032 | -0.612 | 1 | 1 |
